# Analysis of left ventricular rotational deformation by 2D speckle tracking echocardiography: a feasibility study in athletes

**DOI:** 10.1007/s10554-021-02213-3

**Published:** 2021-03-18

**Authors:** Phillipp Hofrichter, Andreas Hagendorff, Ulrich Laufs, Sven Fikenzer, Pierre Hepp, Robert Percy Marshall, Bhupendar Tayal, Stephan Stöbe

**Affiliations:** 1 Department of Cardiology, University Hospital Leipzig, Liebigstrasse 20, 04103 Leipzig, Germany; 2 Division of Arthroscopy, Joint Surgery and Sports Injuries, University Hospital Leipzig, Liebigstrasse 20, 04103 Leipzig, Germany; 3 RasenBallsport Leipzig GmbH, Cottaweg 3, 04177 Leipzig, Germany; 4 Department of Cardiology, University Hospital Aalborg, Hobrovej 18‑22, 9100 Aalborg, Denmark

**Keywords:** 2D speckle tracking, Radial strain, Circumferential strain, Rotation, Twist, Athletes

## Abstract

2D speckle tracking echocardiography (2DSTE) is established to analyse left ventricular (LV) longitudinal function. The analysis of LV rotational deformation is challenging and requires standardization of image acquisition as well as postprocessing analysis. The aim of this study was to test the feasibility to analyse LV rotational deformation using 2DSTE by introducing a novel algorithm for the detection of artefacts. The study was performed in 20 healthy subjects serving as a control group and in 53 competitive sportsmen. Circumferential, radial strain (CS, RS) and LV rotation were analysed by 2DSTE in parasternal short axis views. The stepwise algorithm to exclude potential artefacts starts with the visual estimation of the image quality with respect to complete visualization of all myocardial segments during the entire cardiac cycle followed by the exclusion of data sets in participants with conduction abnormalities. The next step is the optimization of tracking areas and a cross-check of implausible strain waveforms in multiple acquired comparable cineloops. The last step is the exclusion of strain curves with persisting implausible waveforms if standardization failures and incorrect LV wall tracking are fixed. Plausible physiological strain curves were observed in 89% (n = 65/73) of all subjects. In controls all implausible waveforms could be verified as artefacts. The algorithm was applied in 53 professional athletes to test and confirm its feasibility. Abnormal CS waveforms were documented in 25 athletes, verified as artefacts due to tracking failures in 22 athletes and due to incorrect image acquisition in 3 athletes. CS artefacts were mostly located in the basal posterior and lateral LV segments. (endocardial: 6%, n = 4/70; p < 0.05) and basal posterior (endocardial: 8%, n = 5/70; p < 0.05) segments were highly susceptible to artefacts. 2DSTE of parasternal short axis views to analyse circumferential and radial deformation as well as LV rotation is feasible in athletes. The proposed algorithm helps to avoid artefacts and might contribute to standardization of this technique. 2DSTE might provide an interesting diagnostic tool for the detection of viral myocarditis, e.g. in athletes.

**Supplementary Information:**

The online version contains supplementary material available at 10.1007/s10554-021-02213-3.

## Introduction

Over the past decade, the analysis of longitudinal deformation by 2D speckle tracking echocardiography (2DSTE) is established for the assessment of global and regional left ventricular (LV) systolic function with low interobserver variability [1, 2]. This technique is based on tracking of “speckle” displacement in two- and three-dimensional (2D, 3D) frames of the respective echocardiographic cineloops [3]. In addition to global and regional LV longitudinal strain (GLS, RLS), LV radial and circumferential strain (RS, CS) as well as LV rotation have been analyzed and normal ranges have been established [4–9]. However, the feasibility, reproducibility, and accuracy of 2DSTE to analyse LV rotation patterns have been rarely demonstrated and the analysis of RS, CS, LV rotation and twist is still not implemented in clinical routine [10–12].

The main problem of analyzing myocardial mechanics by RS, CS, and LV rotation using 2DSTE is still the necessity of a standard operation procedure for image acquisition and defining tracking areas [11, 13]. Such a standard operation procedure only provides good reproducibility in terms of intra- and interobserver variability to establish 2DSTE into the clinical scenario. Further, even with a standardized acquisition protocol and algorithm for artefact detection, the intervendor variability of 2DSTE has to be considered [14].

The different LV layers contribute to radial contraction by different components of LV deformation. The architecture of the LV myocardial fibers shows longitudinal fibres located predominantly within the inner LV layers and circumferential fibres located predominantly within the outer LV layers, respectively [15, 16]. The synergy of both fibre types results in the systolic twisting and diastolic untwisting, which is important for an energy-efficient LV blood flow by causing the physiological apical vortex formation. In addition, the degree of LV deformation differs between LV apex and LV base as well as between subendo- and subepicardial layers showing a gradient from higher to lower values, respectively [17]. Due to the fibre architecture CS is mainly caused by the outer LV layers. Regarding the fact that the outer third of the LV wall is only contributing with 17% to the total LV wall thickening in comparison to the inner third of the LV wall with 58% a physiological gradient of LV deformation exists, which can be documented by layer-specific 2DSTE [18].

**Fig. 1:**
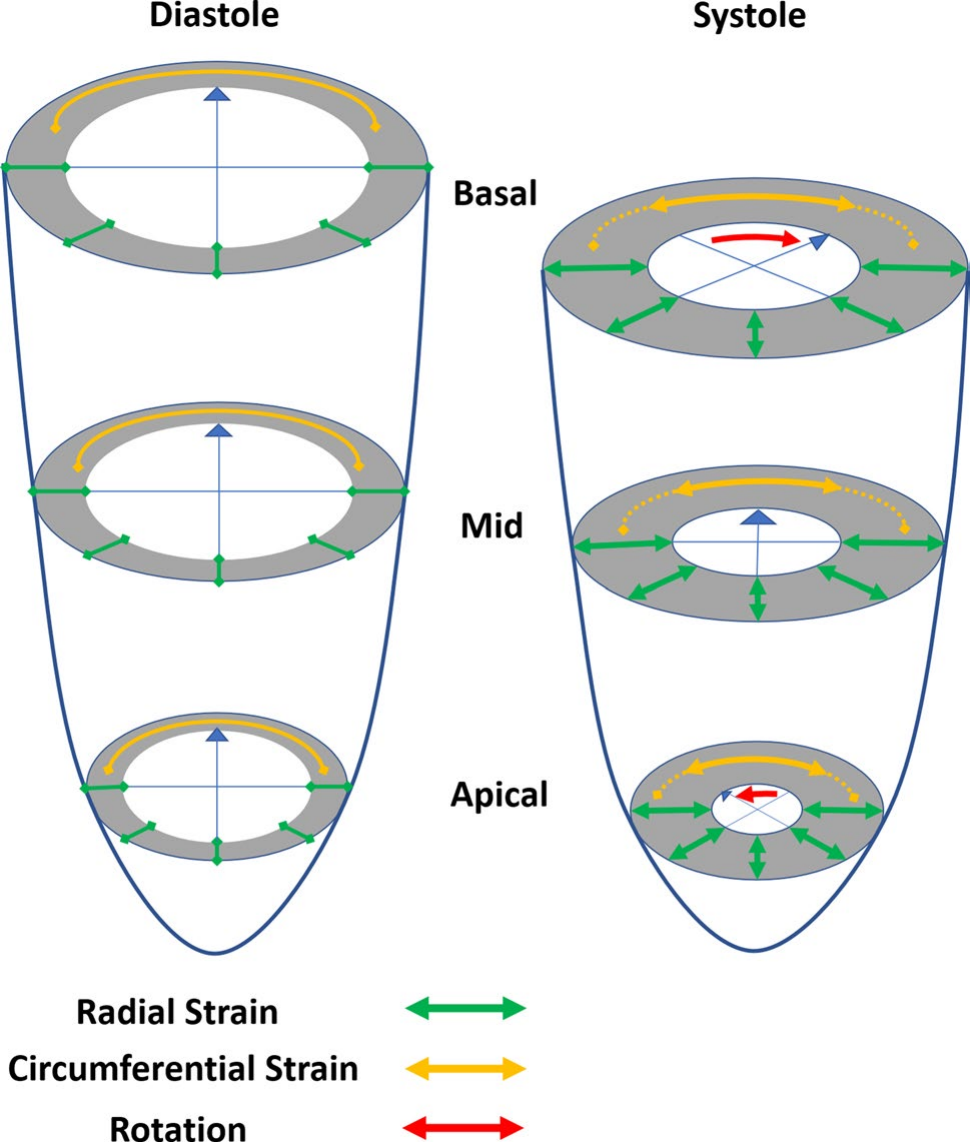
Scheme of the components of left ventricular rotational deformation

The analysis of LV wall motion includes different components of LV rotational deformation (Fig. 1). Standardization of the apical sectional planes can be ensured for GLS and RLS analysis, e. g. by simultaneous triplane image acquisition in 2D transthoracic echocardiography (TTE). In addition, beat-to beat-variability can be avoided by this acquisition modality. Using 2DSTE the analysis of RS, CS and LV rotation is only possible in parasternal short axis views. However, the standardization of parasternal short axis views is more challenging in comparison to apical views.

Optimal parasternal acoustic windows and adequate image quality are of tremendous importance to assess RS, CS an LV rotation by 2DSTE. Consequently, this method of LV strain analysis is preserved for patients, where standardized parasternal image acquisition is possible with adequate image quality. Thus, this 2D technique is predominantly suited for younger, normal weight subjects, e.g. athletes, and patients without obesity and/or pulmonary diseases. The feasibility of 2DSTE, however, should be proven for the analysis of RS, CS, and LV rotation, which might be presumably important for the detection of myocardial involvement in acute myocarditis, which predominantly affects the outer LV layers being responsible for the rotational LV motion [19–25]. To ensure the detection of functional abnormalities of LV rotational deformation due to myocardial inflammation, standardization of image acquisition and postprocessing analysis by 2DSTE is obviously required.

Consequently, the present study focuses (1) on the methodological prerequisites for establishing the analysis of LV rotational deformation in cohorts with adequate parasternal acoustic window and (2) on an algorithm to differentiate pathological findings from artifacts analyzing parasternal short axis views by 2DSTE. Thirdly (3), the feasibility of this approach was tested in a cohort of athletes with the intention to illustrate a new diagnostic option for the detection of myocarditis by echocardiography. The verification of pathological findings and the delineation of pathologies from imaging artifacts are described by a detailed proposed algorithm. The necessity of an adequate acoustic window obviously provides limitations of the described 2DSTE approach. However, the analysis of LV rotational deformation might be helpful in cohorts with normal cardiac geometry, in which adequate image quality of parasternal short axis views is present to detect potential acute myocarditis in future clinical practice.

## Methods

### Participants characteristics

**Fig. 2:**
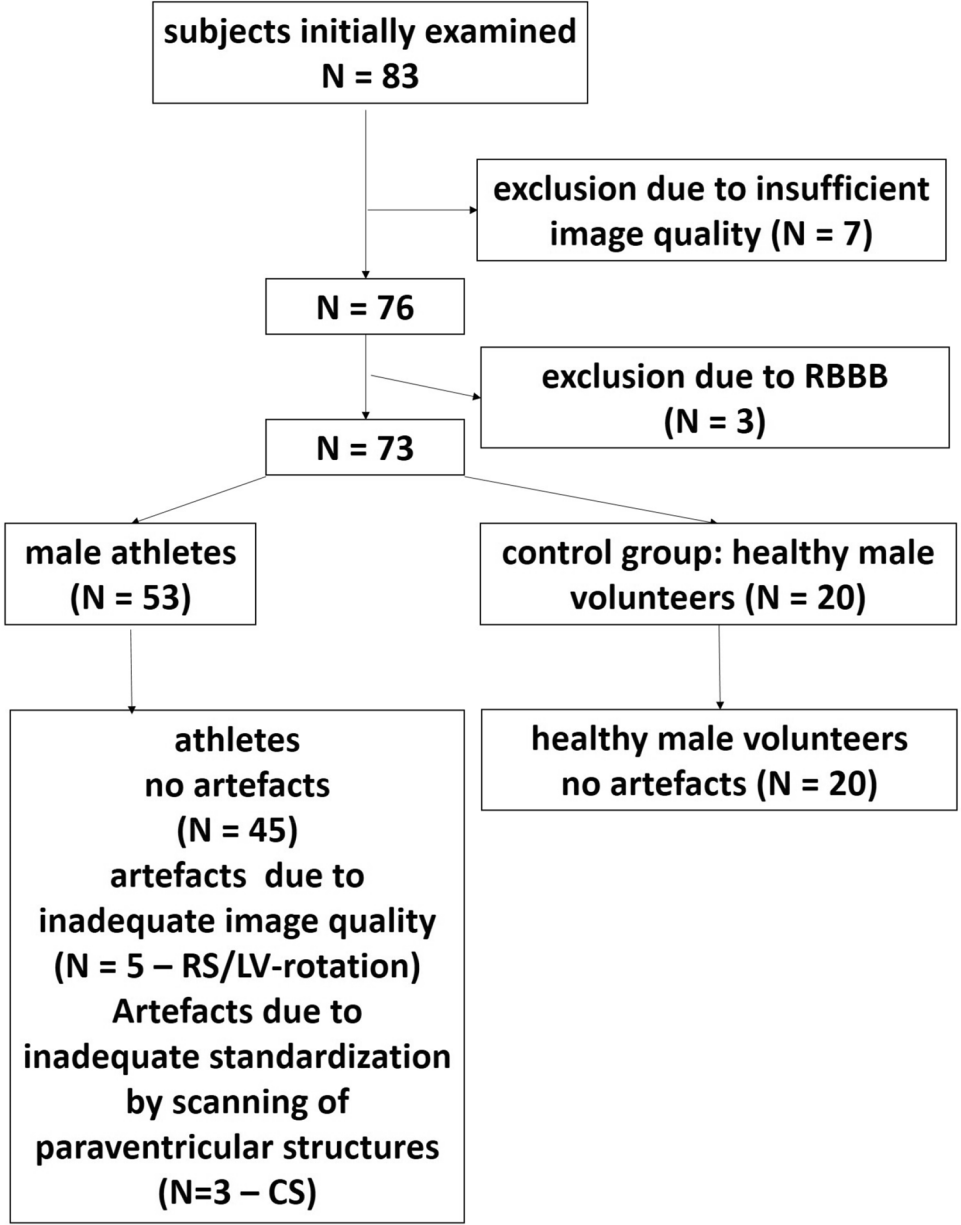
Flow chart of the proposed algorithm. 7 subjects were excluded by the algorithm due to bad image quality by visual estimation, 3 subject were excluded due to abnormal contraction patterns caused by right bundle branch block (RBBB). In 5 athletes all pathological waveforms could be verified as artefacts of radial strain (RS) and/or left ventricular (LV) rotation due to inadequate image. In 3 athletes artefacts could be verified due to imaging failures of oblique sectional planes with visualization of left atrial myocardium, the coronary sinus or the mitral valve annulus

A total of 83 healthy participants (26 controls, 57 athletes) were screened. 5 controls and 2 athletes had to be excluded from the data analysis due to bad parasternal image quality and 3 athletes were excluded due to complete right bundle branch block with regional wall motion abnormalities (Fig. 2). Thus, the study cohort (n = 73) was composed by a group of professional male athletes (PA) (n = 53) and a group of young, healthy male volunteers (n = 20) serving as a control group (CG). The cohort of the controls consists of male students with non-professional sportive activities, the cohort of professional male athletes consists of professional soccer players (n = 25) and handball players (n = 28). Characteristics of the study participants are presented in Table 1. The echocardiography was performed at their annual medical check-up. None of the study subjects had a history of cardiovascular diseases. Blood pressure, heart rate, and electrocardiogram were normal. None of the subjects received any medication or concomitant drugs. All participants were informed in detail about the study and gave individual consent to participate in the study.

**Table 1:** Mean values of participants` characteristics in controls and athletes

Parameters	Control group (n = 20)	Professional athletes (n = 53)	p value
Age (years)	26 ± 3	25 ± 3	0.01*
Height (cm)	180 ± 8	186 ± 7	0.01*
Weight (kg)	78 ± 4	84 ± 5	0.01*
BSA (m^2^)	2.0 ± 0.2	2.1 ± 0.1	0.5
BMI (kg/m^2^)	24 ± 2	24 ± 2	0.9
HR (1/min)	62 ± 9	57 ± 9	0.7
Systolic blood pressure (mmHg)	128 ± 9	124 ± 8	0.9
Diastolic blood pressure (mmHg)	82 ± 11	77 ± 10	0.19
Sports activity (min per week)	–	386 ± 63.5	–
Fat mass (% of weight)	–	11.5 ± 2.0	–

A p-value < 0.05 is considered as statistically significant

### Echocardiographic image acquisition

A standardized 2D TTE protocol was performed (Vivid E9- or Vivid E95, GE Healthcare Ultrasound, Horten, Norway) by two experienced physicians at the University Hospital of Leipzig between June 2019 and May 2020. However, only one echocardiographic investigation was performed in each participant. Prerequisite was generally the documentation of the complete LV wall during the entire cardiac cycle with an optimal image quality of the parasternal short axis views. The short axis views were exactly adjusted perpendicular to the long axis view by previous biplane scanning to avoid oblique LV scanning. In addition, the basal, mid, and apical short axis views were verifiably matched to the respective LV levels of a standardized long axis view by biplane LV scanning and by corresponding apical LV views to enable analysis of LV rotation, twist, and untwisting in relation to maximum end-diastolic and end-systolic LV length. For each sectional plane (apical, mid, and basal short axis view) at least two different loops were recorded to enable a comparison of 2DSTE analysis by postprocessing between the respective cineloops to detect possible contact artefacts between transducer and skin, or artefacts due to rib or lung shadowing.

### Echocardiographic image analysis

Deformation parameters of RS, CS, and LV rotation were assessed by Quantitative Analysis (EchoPAC software v203, GE Healthcare). The tracking areas were placed manually at the epicardial and endocardial contour of the LV wall with at least three different region of interest (ROI) widths for each section. Data were analysed by quantitative assessment of the respective strain graphs.

**Fig. 3:**
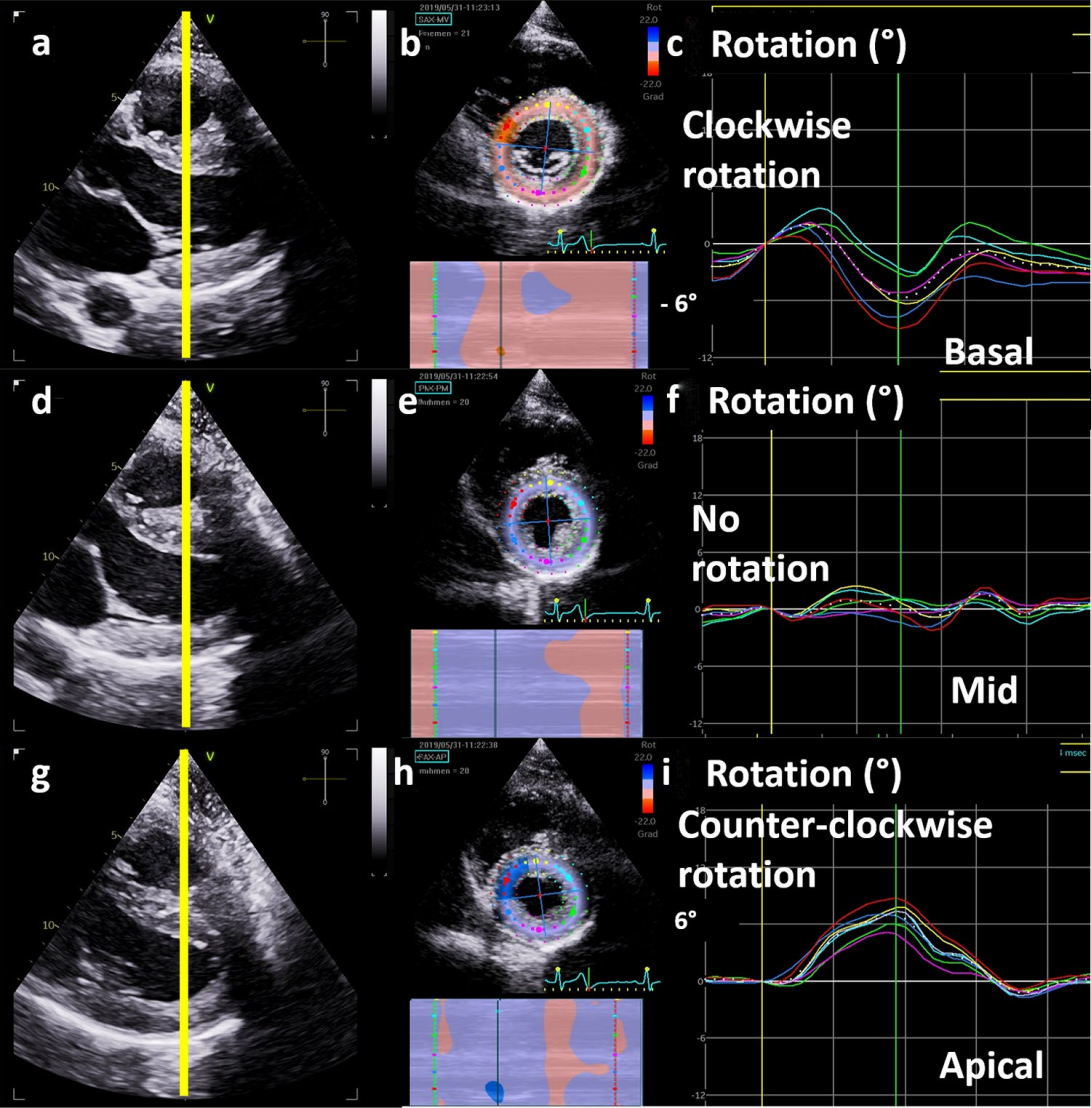
Illustration of physiological waveform patterns of segmental left ventricular rotational strain. Long axis view during systole (**a**) with the corresponding basal short axis view and the respective color M-Mode of regional basal clockwise rotation (**b**) and the corresponding rotation curves (**c**)—the yellow line in (**a**) shows the level of the sectional plane in (**b**); long axis view during systole (**d**) with the corresponding mid short axis view and the respective color M-Mode of regional mid rotation near the zero line (**e**) and the corresponding rotation curves (**f**)—the yellow line in (**d**) shows the level of the sectional plane in (**e**); long axis view during systole (**g**) with the corresponding apical short axis view and the respective color M-Mode of regional apical counter-clockwise rotation (**h**) and the corresponding rotation curves (**i**)—the yellow line in (**g**) shows the level of the sectional plane in (**h**)

**Fig. 4:**
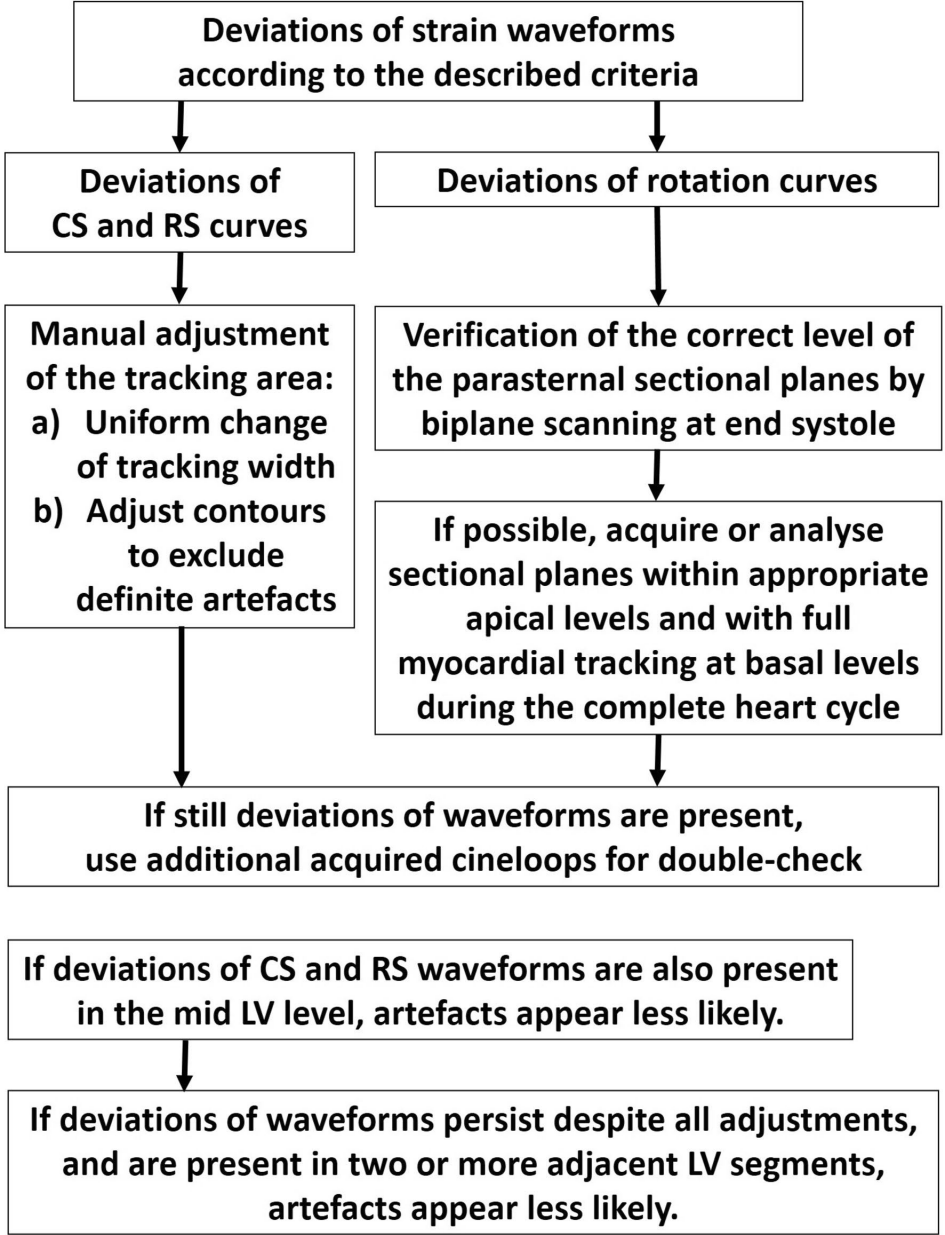
Flow chart of the third step of the algorithm to discriminate artefact from real pathologies of LV deformation. The check of correct speckle tracking is based on the comparison of the speckle tracking analysis in multiple acquired loops and on the manual adjustment of the tracking area

The sequence of the proposed algorithm started with the visual estimation of the image quality with respect to complete visualization of all LV segments. If the LV myocardium of the respective short axis views was not completely visualised during the entire cardiac cycle, images were not accepted, and analysis by 2DSTE is not possible. The next step contained the analysis of strain curves with respect to plausible physiological patterns. Normal waveforms and patterns of RS, CS, and LV rotation curves as well as normal values were defined by the following [4–9]:

1. RS curves show positive monophasic waveforms during systole with a peak maximum at end systole prior to or at aortic valve closure (AVC) reflecting myocardial wall thickness (see on-line Suppl: Fig. 1). Pathological findings are observed in case of maximum RS values after AVC (= post-systolic radial thickening), no significant RS increase or even RS decrease during systole, or dispersion of regional RS curves with maximum values < 25% of the highest maximum value.
2. CS curves show negative monophasic waveforms during systole with a minimum peak at end systole prior to or at AVC reflecting circumferential shortening (see on-line Suppl: Fig. 2). Pathological findings are observed in case of minimum CS values after AVC (= post-systolic circumferential shortening), no significant CS decrease or even CS increase during systole, or dispersion of regional circumferential strain curves with minimum values > 25% of the lowest minimum value.
3. Circumferential layer strain normally shows a homogeneous gradient (delta values) between subendo- and subepicardial strain. Thus, normal CS layer strain is characterized by a homogeneous pattern of reduced subepicardial CS peak values as well as waveformes with lower minimum values in comparison to the subendocardial CS. Pathological findings might be observed if no gradient between subendo- and subepicardial strain, or positive CS values are present (see on-line Suppl: Fig. 3).
4. Basal LV segments rotate clockwise and present a mean rotation of more than − 3° reaching the minimum prior to or at AVC, which corresponds to the mean value of basal LV rotation minus one standard deviation (SD) (Fig. 3). Pathological findings presumably show a mean basal rotation below − 3°, or dispersion of regional apical rotation curves with values about zero or more.
5. Apical LV segments rotate counter-clockwise and present a rotation of more than 3° reaching the maximum prior to or at AVC, which corresponds to the mean value of apical LV rotation minus one SD (Fig. 3). Pathological findings presumably showed a mean apical rotation below 3°, or dispersion of regional apical rotation curves with values about zero or less. However, the lack of apical rotation can also be caused by a far too mid LV position of the parasternal sectional plane.
6. The different direction of basal and apical rotation normally causes LV twisting (systole) and untwisting (diastole) (see on-line Suppl: Fig. 4) [10, 12]. Normal net rotation qualitatively shows by a monophasic shape and by a maximum prior to or at AVC. During diastole only minimum excursions near the zero line are normal. Pathological net twist curves presumably show curves with deviant waveforms.
7. Normal net rotation rate is qualitatively characterized by a biphasic shape and by crossing the zero-line prior to or at AVC. During diastole only minimum excursions near the zero-line are normal.

Deviations of strain waveforms from the described characteristics are present in some cardiac diseases or in case of artefacts showing chaotic/noisy patterns. For the differentiation between artefacts and pathological findings, we propose the third step of the algorithm starting with a second thoroughly reviewing of the short-axis images with respect to standardization failures followed by manual adjustment of the tracking areas to exclude tracking of non-myocardial structures (Fig. 4).

1. If strain waveforms are not normal after tracking analysis by user-independent automated area tracking, the width of the tracking area was adjusted for full myocardial tracking of the LV wall. In a first step, the tracking width was uniformly changed. In a second step, the regional contours of the tracking area were manually corrected within each LV segment.
2. If the complete LV wall is visualized in all segments of the 2D parasternal short axis views, artefacts might be induced by pronounced echo-dense epicardial contours due to paracardial fibrous tissue—especially in the anterior, lateral, and posterior segments—or rib shadowing and lung superimpositions. With respect to these structures, the tracking width was manually adjusted to the width of the respective regional LV myocardium to optimize tracking quality. If strain curves normalize after this postprocessing manoeuvre, the previously abnormal waveforms is presumably an artefact.
3. The analysis of apical rotation was of particular importance. The counter-clockwise rotation of the LV apex is only detectable, if the sectional plane is really within LV regions near the LV apex. The apical LV level of a short axis view can be documented by biplane scanning directly prior to the acquisition of the respective 2D short axis views. The acquired apical sectional plane should be within the apical third of the LV long axis during end systole to ensure the correct visualisation of rotational LV deformation. In addition, biplane scanning is helpful to detect parts of the aortic root, the coronary sinus or the left atrium, if scanning of the basal short axis view is too caudal and not perpendicular to the LV long axis.
4. If pathologic waveforms are still present after these sequences of the algorithm, all these manoeuvres should be applied in additionally acquired cineloops.
5. Findings of adjacent LV regions should be interpreted with respect to the plausibility of pathological findings, e. g. if suspicious findings of mid LV segments can also be documented in the corresponding apical or basal segments, the suspicious findings are highly susceptible for pathological entities.
6. The reproducibility of pathologic waveforms after passing through this algorithm should be checked in all additionally acquired loops of the same sectional plane.

### Statistics

All statistical analyses to compare mean values of the alphanumeric data between controls and athletes were performed using SPSS version 24.0 (SPSS Inc., Chicago, IL, USA). Group difference verification was performed using the Mann–Whitney U test. A p value < 0.05 was considered to indicate statistical significance. Inter-observer variability of all presented parameters was assessed in ten randomly selected controls and ten athletes, respectively, by two experienced investigators using the same datasets blinded to each other’s results as performed in previous studies [21].

## Results

Mean values for RS, CS, and LV rotation after adjusting the tracking area to avoid artefacts determined in healthy volunteers and athletes are presented in Table 2. Mean values of regional CS of both groups are presented in Table 3, of regional RS in Table 4, and of regional LV rotation in Table 5. The inter-observer variability of all measurements was not statistically different, with a coefficient of variation < 5%. In addition, there was no difference between both investigators regarding the frequency of detecting artefacts using the described algorithm (Table 6—CS inter-observer variability).

**Table 2:** Mean values of basal, mid, and apical circumferential and radial strain as well as mean values of left ventricular rotation in controls and athletes. In addition, the mean ∆ is presented. (no significant differences were observed; p-values < 0.05 are considered as statistically significant)

		Control Group (n = 20)	Professional athletes (n = 50)	Mean ∆ ± *SD*	p-value
		GS value ± *SD*	GS value ± *SD*		
Circumferential strain	Basal	− 26.56 ± 5.32	− 29.67 ± 6.32	3.11 ± 1.24	0.08
	Mid	− 26.13 ± 5.88	− 27.79 ± 5.55	1.66 ± 0.48	0.15
	Apical	− 26.93 ± 6.25	− 31.65 ± 5.35	4.72 ± 1.25	0.06
Radial strain	Basal	28.19 ± 6.65	30.02 ± 6.20	1.83 ± 0.64	0.18
	Mid	29.23 ± 6.28	29.91 ± 4.21	0.68 ± 0.26	0.3
	Apical	31.77 ± 7.73	31.38 ± 4.10	0.39 ± 0.12	0.3
Rotation	Basal	6.32 ± 3.27	5.19 ± 3.59	1.13 ± 0.67	0.1
	Mid	0.92 ± 2.83	0.58 ± 3.20	0.34 ± 0.29	0.09
	Apical	− 5.93 ± 3.82	− 6.7 ± 3.01	0.77 ± 0.25	0.09

**Table 3:** Mean values of basal, mid, and apical regional circumferential strain of the subepicardial, full myocardial, and subendocardial layers in controls and athletes. In addition, the mean ∆ is presented. (no significant differences were observed; p-values < 0.05 are considered as statistically significant)

Circumferential strain			Control Group	Professional athletes	Mean ∆ ± *SD*	p-value
			(n = 20)	(n = 50)		
			GS value ± *SD*	GS value ± *SD*		
Sub-epicardial	Basal	Anterior	− 19.82 ± *4.75*	− 17.38 ± *3.63*	2.44 ± *1.02*	> *0.1*
		Lateral	− 15.28 ± *4.69*	− 14.7 ± *3.21*	0.58 ± *0.23*	> *0.1*
		Posterior	− 12.16 ± *3.56*	− 15.58 ± *4.39*	3.42 ± *1.72*	*0.07*
		Inferior	− 12.89 ± *4.34*	− 14.8 ± *4.22*	1.91 ± *1.01*	> *0.1*
		Septal	− 15.5 ± *3.05*	− 13.42 ± *4.43*	2.08 ± *1.51*	> *0.1*
		Anteroseptal	− 19.3 ± *4.51*	− 18.18 ± *4.28*	1.12 ± *0.39*	> *0.1*
	Mid	Anterior	− 16.42 ± *5.12*	− 18.93 ± *4.84*	2.51 ± *1.43*	> *0.1*
		Lateral	− 20.05 ± *6.34*	− 19.55 ± *4.39*	0.50 ± *0.43*	> *0.1*
		Posterior	− 15.95 ± *7.93*	− 14.63 ± *4.43*	1.32 ± *0.69*	> *0.1*
		Inferior	− 23.9 ± *4.92*	− 22.46 ± *3.05*	1.44 ± *0.56*	> *0.1*
		Septal	− 17.12 ± *4.27*	− 18.93 ± *4.64*	1.81 ± *0.34*	> *0.1*
		Anteroseptal	− 18.81 ± *4.32*	− 17.9 ± *3.56*	0.91 ± *0.15*	> *0.1*
	Apical	Anterior	− 14.93 ± *5.10*	− 14.1 ± *4.34*	0.83 ± *0.51*	> *0.1*
		Lateral	− 16.1 ± *5.81*	− 14.5 ± *3.05*	1.60 ± *1.12*	> *0.1*
		Posterior	− 16.13 ± *4.28*	− 18.2 ± *4.51*	2.07 ± *1.34*	> *0.1*
		Inferior	− 17.43 ± *4.84*	− 18.57 ± *5.12*	1.14 ± *0.93*	> *0.1*
		Septal	− 17.78 ± *4.39*	− 17.43 ± *6.34*	0.35 ± *0.92*	> *0.1*
		Anteroseptal	− 16.68 ± *4.13*	− 19.71 ± *7.93*	3.03 ± *1.27*	*0.08*
Mid = full-myocardial	BASAL	Anterior	− 27.77 ± *4.43*	− 28.08 ± *4.43*	0.31 ± 0.02	> *0.1*
		Lateral	− 26.72 ± *5.69*	− 31.39 ± *5.69*	4.67 ± 1.23	*0.06*
		Posterior	− 25.35 ± *3.56*	− 28.93 ± *4.39*	3.58 ± 1.72	> *0.1*
		Inferior	− 25.47 ± *4.34*	− 31.91 ± *4.22*	6.44 ± 2.01	*0.06*
		Septal	− 26.83 ± *3.05*	− 24.52 ± *4.43*	2.31 ± 1.51	> *0.1*
		Anteroseptal	− 26.57 ± *4.51*	− 28.58 ± *4.28*	2.01 ± 0.39	> *0.1*
	Mid	Anterior	− 26.14 ± *5.12*	− 31.27 ± *4.84*	5.13 ± 1.43	0.07
		Lateral	− 24.98 ± *6.34*	− 25.86 ± *4.39*	0.88 ± 0.43	> *0.1*
		Posterior	− 29.57 ± *7.93*	− 29.12 ± *4.43*	0.45 ± 0.19	> *0.1*
		Inferior	− 27.1 ± *4.92*	− 25.33 ± *3.05*	1.77 ± 0.56	> *0.1*
		Septal	− 28.82 ± *4.27*	− 27.38 ± *4.64*	1.44 ± 0.34	> *0.1*
		Anteroseptal	− 25.69 ± *4.32*	− 27.27 ± *3.56*	1.58 ± 1.05	> *0.1*
	Apical	Anterior	− 26.1 ± *5.10*	− 29.8 ± *4.34*	3.70 ± 1.51	> *0.1*
		Lateral	− 25.68 ± *5.81*	− 25.56 ± *3.05*	0.12 ± 0.12	> *0.1*
		Posterior	− 27.12 ± *4.28*	− 34.8 ± *4.51*	7.68 ± 1.34	*0.08*
		Inferior	− 27.61 ± *4.84*	− 33.2 ± *5.12*	5.59 ± 1.93	> *0.1*
		Septal	− 25.81 ± *4.39*	− 29.54 ± *6.34*	3.73 ± 1.92	> *0.1*
		anteroseptal	− 27.77 ± *4.43*	− 30.52 ± *7.93*	2.75 ± 1.27	> *0.1*
Sub-endocardial	Basal	Anterior	− 35.72 ± *4.41*	− 38.78 ± *4.05*	3.06 ± *1.02*	*0.06*
		Lateral	− 39.16 ± *5.98*	− 48.08 ± *5.03*	8.92 ± *2.23*	*0.06*
		Posterior	− 38.54 ± *3.56*	− 42.28 ± *4.39*	3.74 ± *1.72*	*0.08*
		Inferior	− 38.05 ± *4.34*	− 47.02 ± *4.22*	8.97 ± *3.01*	*0.07*
		Septal	− 38.16 ± *3.05*	− 37.62 ± *4.43*	0.54 ± *0.51*	> *0.1*
		Anteroseptal	− 33.84 ± *4.51*	− 38.98 ± *4.28*	5.14 ± *2.39*	0.07
	Mid	Anterior	− 38.86 ± *5.12*	− 43.61 ± *4.84*	4.75 ± *2.43*	> *0.1*
		Lateral	− 29.91 ± *6.34*	− 32.17 ± *4.39*	2.26 ± *2.43*	> *0.1*
		Posterior	− 43.19 ± *7.93*	− 43.61 ± *4.43*	0.42 ± *0.69*	> *0.1*
		Inferior	− 30.3 ± *4.92*	− 28.2 ± *3.05*	2.10 ± *1.56*	> *0.1*
		Septal	− 40.52 ± *4.27*	− 35.83 ± *4.64*	4.69 ± *1.34*	> *0.1*
		Anteroseptal	− 32.57 ± *4.32*	− 36.64 ± *3.56*	4.07 ± *1.05*	> *0.1*
	Apical	Anterior	− 37.27 ± *5.10*	− 45.5 ± *4.34*	8.23 ± *2.51*	> *0.1*
		Lateral	− 35.26 ± *5.81*	− 36.66 ± *3.05*	1.40 ± *0.12*	> *0.1*
		posterior	− 38.11 ± *4.28*	− 41.38 ± *4.51*	3.27 ± *1.34*	> *0.1*
		Inferior	− 37.79 ± *4.84*	− 43.83 ± *5.12*	6.04 ± *1.42*	*0.06*
		Septal	− 33.84 ± *4.39*	− 41.65 ± *6.34*	7.81 ± *1.92*	> *0.1*
		Anteroseptal	− 39.86 ± *4.76*	− 41.33 ± *7.16*	1.47 ± *0.27*	> *0.1*

**Table 4:** Mean values of basal, mid, and apical regional radial strain in controls and athletes. In addition, the mean ∆ is presented. (no significant differences were observed; p-values < 0.05 are considered as statistically significant)

Radial strain		Control group	Professional athletes	Mean ∆ ± SD	p-value
		(n = 20)	(n = 50)		
		GS value ± SD	GS value ± SD		
Basal	Anterior	27.66 ± 4.96	31.19 ± 4.43	3.53 ± 1.32	0.07
	Lateral	28.25 ± 5.12	34.96 ± 3.08	6.71 ± 1.10	0.06
	Posterior	27.96 ± 3.64	30.78 ± 3.56	2.82 ± 1.81	0.08
	Inferior	28.41 ± 4.13	27.81 ± 4.34	0.60 ± 0.28	0.07
	Septal	32.62 ± 3.05	37.12 ± 3.15	4.5 ± 1.84	> 0.1
	Anteroseptal	32.16 ± 2.99	30.61 ± 4.51	1.55 ± 0.39	0.07
Mid	Anterior	26.62 ± 3.85	34.17 ± 5.12	7.55 ± 2.43	> 0.1
	Lateral	28.16 ± 4.11	35.92 ± 6.34	7.76 ± 1.34	> 0.1
	Posterior	28.30 ± 5.10	35.37 ± 7.93	7.07 ± 1.15	> 0.1
	Inferior	29.14 ± 5.81	33.45 ± 4.92	4.31 ± 1.51	> 0.1
	Septal	30.99 ± 4.28	36.72 ± 4.27	5.73 ± 2.12	> 0.1
	Anteroseptal	29.01 ± 4.84	31.18 ± 4.32	2.17 ± 0.34	> 0.1
Apical	Anterior	33.88 ± 5.69	36.81 ± 5.10	2.93 ± 1.43	> 0.1
	Lateral	39.63 ± 3.56	41.75 ± 5.81	2.12 ± 0.92	> 0.1
	Posterior	37.36 ± 4.34	38.45 ± 4.28	1.09 ± 0.27	> 0.1
	Inferior	25.43 ± 3.05	33.54 ± 4.84	8.11 ± 1.32	0.08
	Septal	32.67 ± 4.51	41.32 ± 4.37	8.65 ± 2.39	> 0.1
	Anteroseptal	26.5 ± 5.12	34.18 ± 2.89	7.68 ± 1.43	> 0.1

**Table 5:** Mean values of basal, mid, and apical regional left ventricular rotation in controls and athletes. In addition, the mean ∆ is presented. (no significant differences were observed; p-values < 0.05 are considered as statistically significant)

Rotation		Control group	Professional athletes	Mean ∆ ± SD	p-value
		(n = 20)	(n = 50)		
		Mean value ± SD	Mean value ± SD		
Basal	Anterior	6.09 ± 2.34	5.29 ± 2.64	0.8 ± 0.43	> 0.1
	Lateral	4.15 ± 2.69	7.69 ± 2.56	3.54 ± 0.69	> 0.1
	Posterior	7.92 ± 2.56	7.54 ± 2.34	0.38 ± 0.56	> 0.1
	Inferior	7.43 ± 2.34	5.71 ± 3.05	1.72 ± 0.34	> 0.1
	Septal	4.73 ± 3.15	4.95 ± 2.51	0.22 ± 0.05	> 0.1
	Anteroseptal	5.37 ± 2.51	7.51 ± 2.12	2.14 ± 0.51	> 0.1
Mid	Anterior	− 0.01 ± 1.12	0.13 ± 1.32	0.14 ± 0.12	> 0.1
	Lateral	0.65 ± 1.34	0.31 ± 1.02	0.34 ± 1.34	> 0.1
	Posterior	0.24 ± 2.01	− 0.06 ± 0.82	0.3 ± 0.09	> 0.1
	Inferior	0.14 ± 0.92	0.87 ± 0.46	0.73 ± 0.92	> 0.1
	Septal	0 ± 1.27	0.25 ± 1.02	0.25 ± 1.27	> 0.1
	Anteroseptal	− 0.03 ± 0.92	0.14 ± 0.65	0.17 ± 0.07	> 0.1
Apical	Anterior	− 5.79 ± 3.10	− 7.5 ± 2.10	1.71 ± 1.10	> 0.1
	Lateral	− 5.55 ± 2.81	− 6.9 ± 3.92	1.35 ± 0.43	> 0.1
	Posterior	− 5.32 ± 2.34	− 6.4 ± 2.89	1.08 ± 0.28	> 0.1
	Inferior	− 7.31 ± 3.69	− 8.03 ± 3.05	0.72 ± 0.84	> 0.1
	Septal	− 7.93 ± 2.6	− 7.35 ± 3.48	0.58 ± 0.39	> 0.1
	Anteroseptal	− 5.22 ± 2.43	− 6.03 ± 2.46	0.81 ± 0.97	> 0.1

**Table 6:** Inter-observer variability of basal, mid, and apical circumferential strain assessed in ten controls and ten athletes

	Observer 1	Observer 2	p-value
Detected artefact Total data sets (n = 20)	5/20	6/20	0.1
Circumferential strain			
Basal	− 25.91 ± *2.49*	− 26.23 ± *3.69*	> 0.1
Mid	− 26.58 ± *3.11*	− 27.78 ± *2.81*	> 0.1
Apical	− 27.31 ± *3.27*	− 26.93 ± *2.39*	> 0.1

**Fig. 5:**
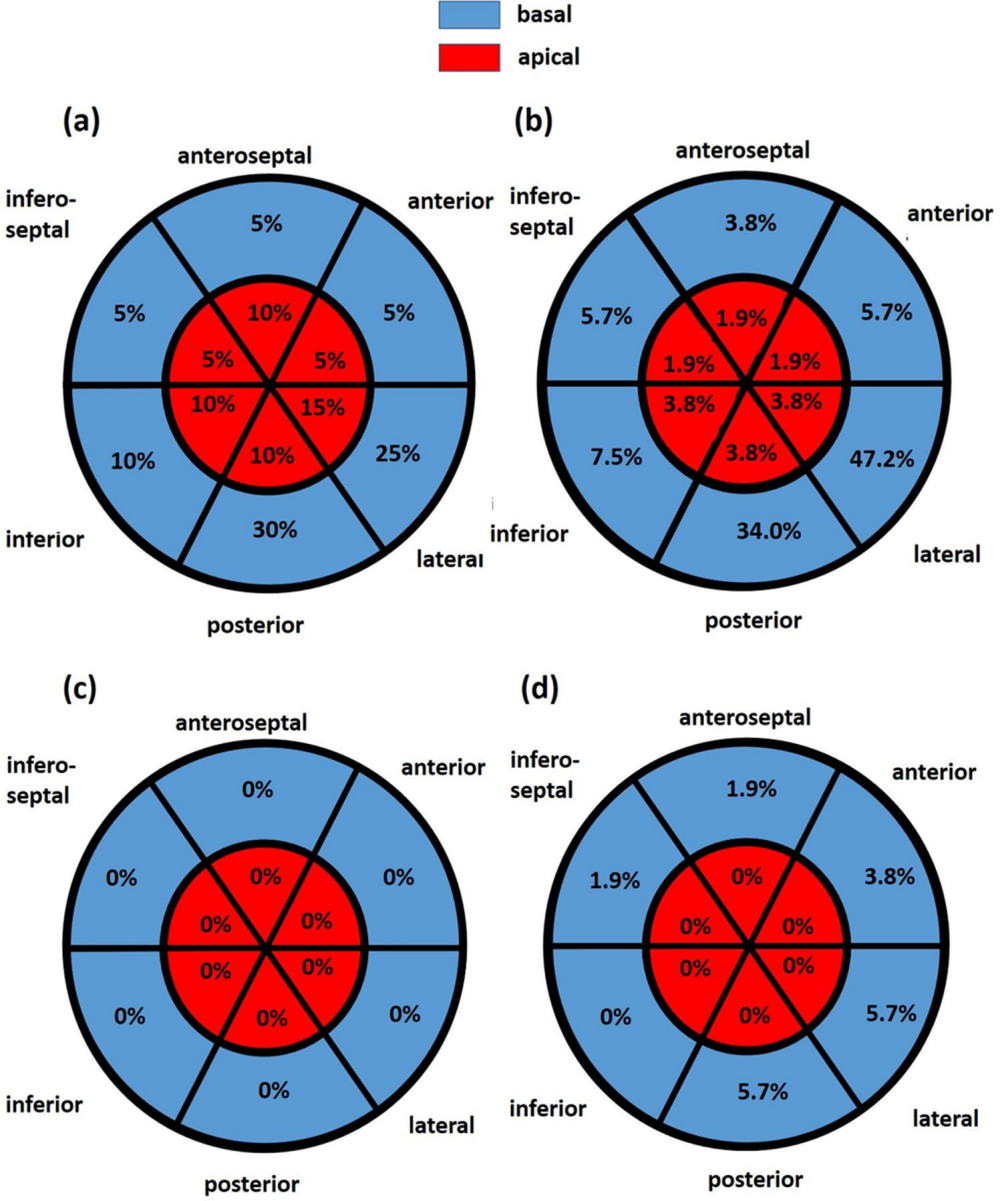
Illustration of the frequency of artefacts assessed by circumferential strain analysis within the respective basal and apical left ventricular (LV) segments by automated full myocardial tracking in healthy volunteers (**a**) and professional athletes (**b**). It is obvious that the basal posterior and lateral LV segments are most prone to artefact tracking. Illustration of the frequency of artefacts of circumferential strain analysis assessed within the respective basal and apical left ventricular segments after manual adjustment of the tracking area in healthy volunteers (**c**) and professional athletes (**d**). In controls no artefact tracking was observed. In athletes all residual deviations of strain waveforms could be classified as artefacts due to failure of standardization during image acquisition

CS abnormal strain waveforms was most frequently observed. In 6 of 20 controls and in 25 of the athletes abnormal CS findings were present (Fig. 5). All these findings were identified as artefacts by applying the proposed algorithm, either by optimizing the tracking area (n = 6 in controls; n = 22 in athletes) or by identifying paraventricular structures as causes for the abnormal waveforms (n = 3 in athletes). In 41 of 73 subjects (56%) at least one waveform was defined as pathologic for RS, CS, or LV rotation. Thus, in all cases with abnormal CS waveforms and in 10 additional athletes, RS or LV rotation was abnormal. In these 10 cases the abnormalities were caused by physiological entities like mitral annulus disjunction causing early systolic CS lengthening or RS thinning or by aberrant chordae in the LV apex causing post-systolic shortening of apical contraction, by inadequate image quality of the apical sectional planes due to pulmonary interferences, or acquisition of sectional planes within the mid third of the left ventricle ruling out reliable analysis of rotational deformation. Thus, after adjustment of the tracking area by the proposed algorithm the number of pathological waveforms decreased significantly (p < 0.05). No abnormalities were observed in the control group, and only 8 of 53 athletes (11%) showed remaining abnormal abnormalities, which could be explained after applying the proposed algorithm.

### Detection of radial strain artefacts

Pathological regional RS resulted in regional pathological waveforms presenting postsystolic RS maxima after AVC, reduced or missing peak maximum values or even negative values during systole. Pathological RS patterns were observed for apical, mid as well as basal sectional short axis views in 4 individuals, who also presented pathological CS waveforms. Obviously, after adjusting the tracking area according to the proposed algorithm, the pathological RS waveforms were eliminated or could be defined as artefacts due to tracking of intersected atrial myocardium, coronary sinus, or the aortic root.

**Fig. 6:**
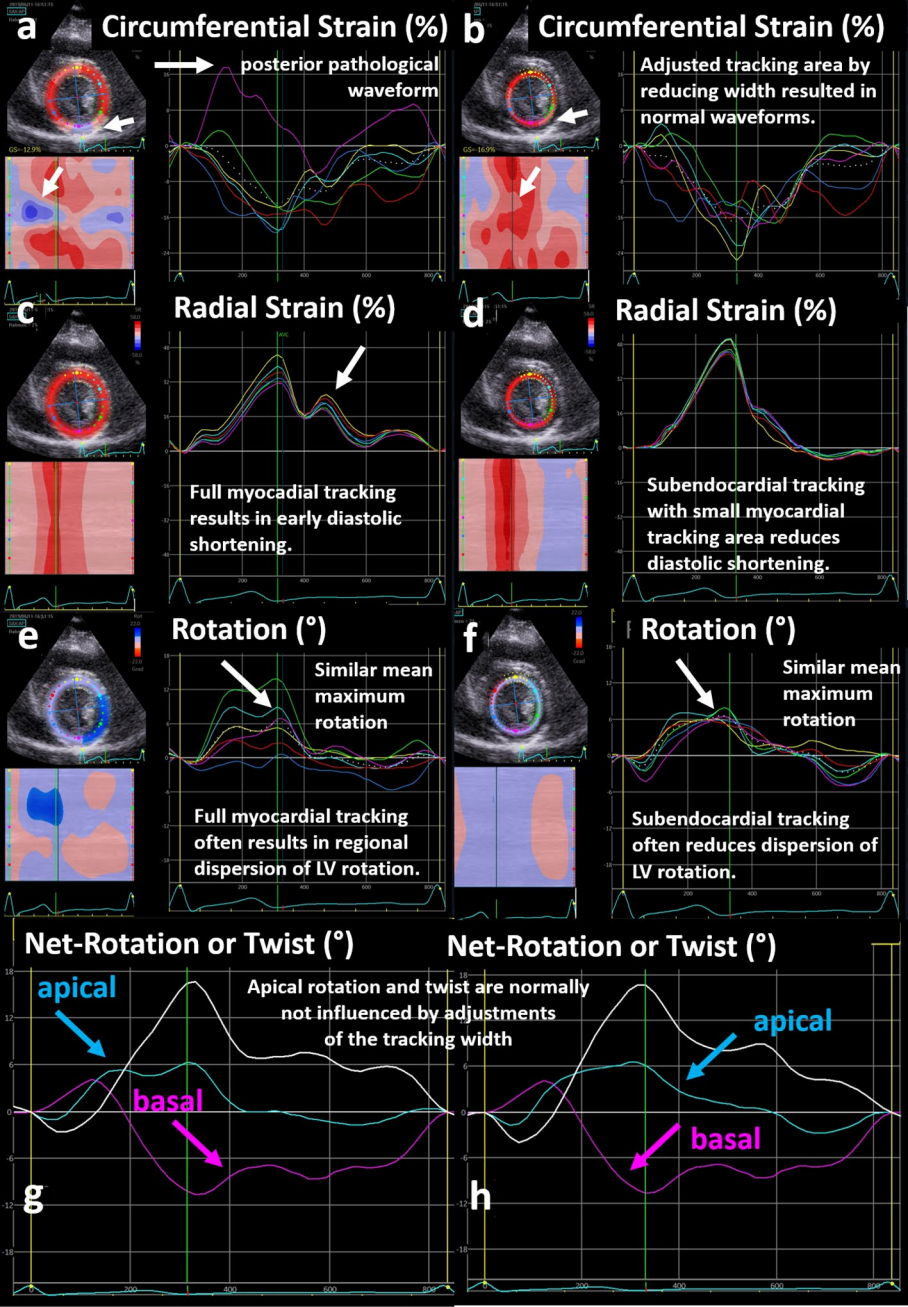
Illustration of alterations of left ventricular (LV) strain and rotation patterns with reduction of the width of the tracking area and correct endocardial border tracking. On the left side full myocardial tracking is displayed, on the right side predominantly subendocardial tracking. Full myocardial tracking often results in artefact tracking of circumferential strain in the posterior regions due to tracking of pericardial structures during systole (**a**), which can be checked by reducing the tracking width in this region as shown by subendocardial tracking (**b**). Full myocardial tracking of radial strain often results in post-systolic diastolic shortening and reduced peak maximum values (**c**) in comparison to subendocardial tracking with higher peak maximum values (**d**). Full myocardial tracking of LV rotation often results in a dispersion of the regional rotation curves (**e**). Subendocardial tracking results in similar mean maximum rotation values (**f**). Net rotation results in similar curves (**g**, **h**) because mean values of basal and apical rotation do not significantly differ with different tracking width of the tracking area. However, the prerequisite is to avoid tracking of non-myocardial structures

The width of the tracking area and its position to subendo- or subepicardial layers influence the corresponding strain waveforms (Fig. 6). If the width of the tracking area was reduced while maintaining the endocardial borderline, the CS minima decreased, the RS maxima increased, and the dispersion of regional LV rotation was reduced (Fig. 6). However, if apical parasternal short axis views were adequate, the width of the tracking area had no influence on net-rotation and net-rotation rate.

### Detection of circumferential strain artefacts

Pathological waveforms of regional CS showed different distributions depending on LV segments. The most frequent LV segments, in which pathological CS were determined, were the basal lateral (CG = 25%, 5/20; PA = 35.7%, 25/70), basal posterior (CG = 30%, 6/20; PA = 25.7%, 18/70), apical lateral, apical posterior, and apical inferior LV segment (CG = 15%, 10%, 10%; 3/20, 2/20, 2/20; PA = 2.9% each, 2/70) using automated tracking areas without manual correction (Fig. 5). No significant difference between the control group and professional athletes were observed.

After adjustment of the tracking areas by the proposed algorithm the frequency and distribution of regional pathological waveforms significantly decreased in both, apical and basal LV segments (p < 0.05). However, pathological CS waveforms were still observed in the basal lateral and basal posterior LV segment (CG = 5%, 1/20; PA = 4.3%, 3/70 respectively). All these pathological CS waveforms could be allocated to artefacts due to scanning of structures beside the LV wall, which were caused by inadequate sectional planes. No pathological waveforms have been documented in the apical LV segments after following the proposed algorithm (Fig. 5). Two main causes of artefacts could be identified. Firstly, air- and rib shadowing as well as bad transducer to skin contact (see on-line Suppl: Fig. 5); secondly, the tracking of LV structures beside the LV myocardium—mostly due to incorrect sectional planes (see on-line Suppl: Fig. 6).

The adjustment of the tracking area using the proposed algorithm erased all observed pathological CS waveforms in adequate LV myocardial tracking. The risk of tracking failure due to extreme narrowing of the tracking area was more pronounced for analysis of RS than of CS (Fig. 4). After manual adjustment of the tracking area using the proposed algorithm 4 of 70 subjects (5.7%) still presented pathological waveforms.

### Detection of LV rotation artefacts

**Fig. 7:**
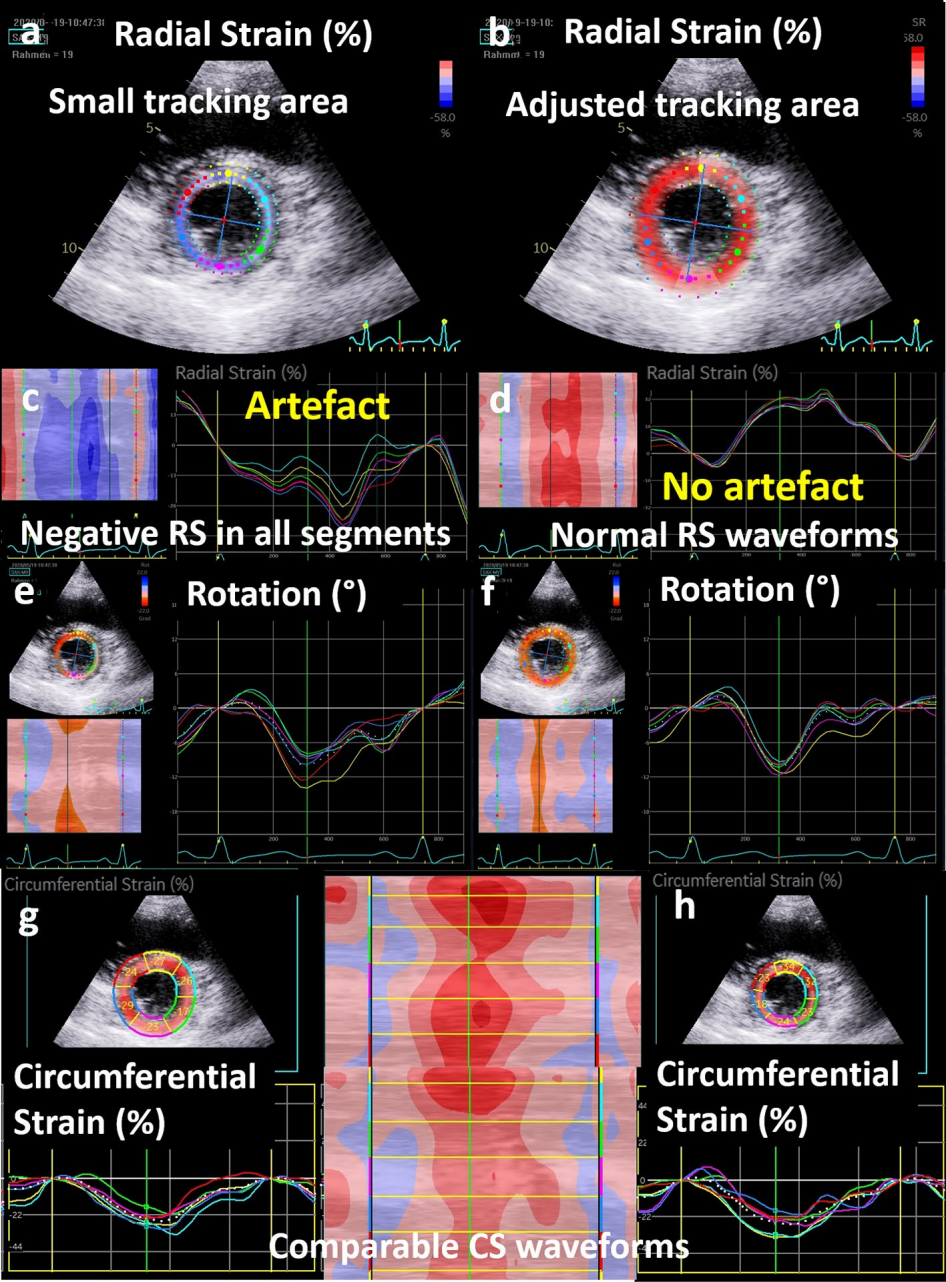
Illustration of artefact tracking due to wrong labelling of the tracking area. A relatively too small tracking area with endocardial contours within the cardiac cavity is displayed in (**a**), an adjusted full myocardial tracking area in the same cineloop in (**b**). The wrong labelling of the tracking area resulted in negative radial strain patterns (**c**) which can obviously identified as an artefact. Adjustment of the tracking area resulted in physiological waveforms (**d**). Surprisingly, the corresponding curves of left ventricular rotation (**e**, **f**) and circumferential strain (**g**, **h**) showed only minor differences with still physiological waveforms

The magnitude of LV rotation mainly depends on the LV level, in which LV rotation is analysed. Basal LV rotation is presumably normal, if the mean value of maximum peak systolic rotation is more than − 3°; apical LV rotation is presumably normal, if the mean value of maximum peak systolic rotation is more than 3°. The mean values of LV rotation determined in all basal and apical rotation are − 5.93° ± 3.82° and 6.32° ± 3.27°, respectively. However, if apical LV rotation the apical LV third is assumed within this range > 3°, still 10% of the values (7 of 73) were below this range in the present study (see on-line Suppl: Fig. 7). The dispersion of regional LV rotation often varying with changing width of the tracking area, whereas the corresponding mean value of LV rotation and the waveform of global LV rotation remained in the same range (Figs. 7). The dependency of the dispersion of regional LV rotation on the width of the tracking area was more pronounced in apical in comparison to mid or basal LV levels (Fig. 7). The more apical the short axis view is recorded, the higher maximum counter-clockwise rotation was observed, which presumably reflect the physiological conditions (see on-line Suppl: Fig. 7).

**Fig. 8:**
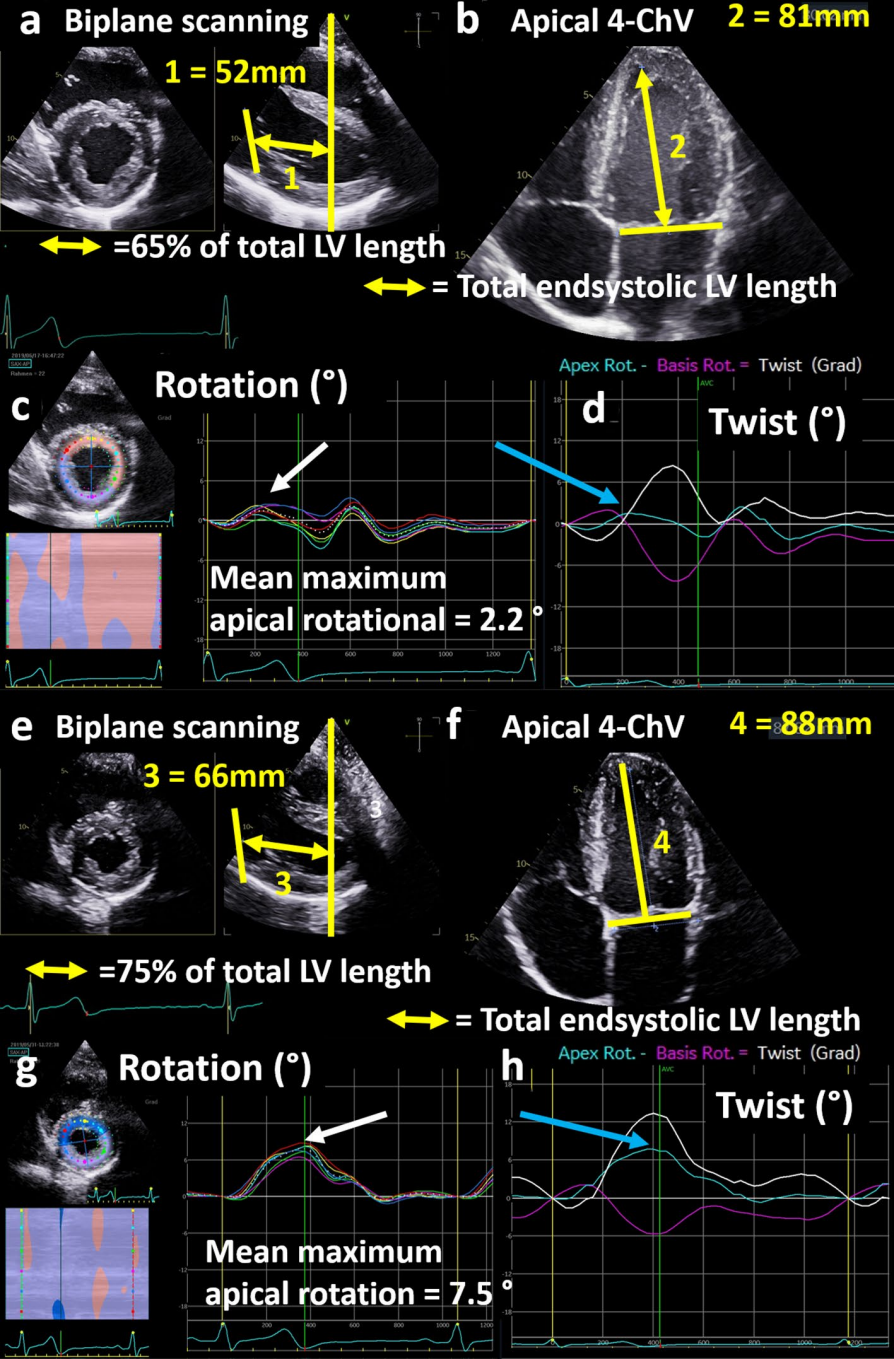
Illustration of standardization of the apical short axis view for verifiable analysis of apical rotation: biplane scanning documents a distance of 52 mm (1) between the mitral annulus and the respective level of the short axis view at end systole in (**a**). The total endsystolic length of the left ventricle documented in the 4-chamber view (4-ChV) is 81 mm (2) (**b**). Thus, the short axis level is in the range of 65% of the total left ventricular (LV) length representing a midventricular short axis view. In consequence, LV rotation is near the zero line (**c**) and the net-rotation or twist curve is misleading (**d**—blue arrow) because apical rotation was incorrectly assessed. In contrast, biplane scanning documents a distance of 66 mm (3) between the mitral annulus and the respective level of the short axis view at end systole in (**e**). The total endsystolic length of the left ventricle documented in the 4-ChV is 88 mm (4) (**f**). Thus, the short axis level in this example is in the range of 75% of the total LV length representing an apical short axis view. In consequence, the LV rotates counter-clockwise (**g**) and the net-rotation or twist curve is plausible (**h**—blue arrow) because apical rotation was correctly assessed. Thus, it is crucial to document the correct level of the short axis view to interpret data of LV twist and untwisting

The apical short axis views can be checked to represent a sectional plane within the apical regions (Fig. 8). To detect reliable maximum values of the apical LV rotation the acquired apical short axis view should be within the apical third of the mid LV long axis determined in the apical views (Fig. 8). In apical short axis views within this range all peak maxima of apical LV rotation had almost been higher than 3° (see on-line Suppl: Fig. 7).

## Discussion

The main findings of the study were that the proposed algorithm verifies artefacts by about 90% and strain analysis did not show different results in controls and athletes. CS artefacts are most frequently observed in the basal lateral and basal posterior LV segments caused by inadequate sectional planes through the atrial myocardium, the coronary sinus and/or the mitral valve annulus. RS and LV rotation artefacts are mostly due to limited image quality caused by rib shadowing and lung superimpositions. A reliable LV rotation analysis including LV twist and untwisting is only possible, if the apical short axis views are acquired within the apical LV quarter and the basal short axis view within the basal LV quarter. To the best of our knowledge, the current study is the first to evaluate the feasibility of 2DSTE in a selected cohort with adequate parasternal acoustic window.

### The rationale to test the feasibility of 2DSTE analysis of LV rotational deformation in athletes

2DSTE is in principle able to analyse the complex morpholocical and functional interactions between the LV fibres orientation and LV deformation [26–30]. However, one important requirement of 2DSTE is to satisfy adequate image quality. Thus, cohorts with excellent image quality favour analysis of LV and right ventricular (RV) function by this method [31–33]. It was convincingly documented, that 2DSTE is suitable to assess physiological adaptation of the LV and RV function to exercise conditioning [31] and to assess the extent and physiological determinants of RV remodeling in highly trained athletes [32, 33]. In addition, LV rotation and torsion assessed by 2DSTE were also defined as determinants in competitive sportsmen to distinguish between athlete’s heart and hypertensive or hypertrophic cardiomyopathy [34, 35]. The role of LV twist in cardiac adaptation of athlete`s heart in endurance sport was also analyzed by 2DSTE [36].

### The rationale to propose an algorithm for the 2DSTE analysis of LV short axis views

Clinical cases presented pathological 2DSTE findings in various diseases documenting the potential use of analyzing RS, CS, LV rotation and twist to detect myocardial pathologies [17, 19, 20, 37, 38]. With respect to this challenge high requirements on image quality have to be considered beside standardization of image acquisition [11, 13]. Thus, an algorithm to detect real pathologies should be robust to detect artefacts due to wrong sectional planes as well as due to wrong tracking manoeuvres. The proposed algorithm for the standardized image acquisition of short axis views highlights the biplane scanning to document the correct short axis view perpendicular to the central LV axis prior to acquisition of the correct monoplane short axis view. The biplane documentation enables an easier detection of paraventricular structures within the standardized short axis views during the cardiac cycle.

The proposed algorith starts with the obvious primary decision to reject the tracking analysis due to bad image quality or due to wrong sectional planes. However, sometimes it is not easy to detect artefacts due to regional failure of tracking quality. In these cases suspicous strain waveforms should be verified and controlled by adjusting the tracking areas as well as by analyzing additional acquired cineloops of the same sectional plane. Thus, the proposed algorithm integrates both, the verification of the image quality and the adjustment of the tracking areas.

The most important issue to focus for RS and CS analysis is the correct choice of the tracking width (Figs. 6 and 7) and the correct delineation of the endocardial contour. Especially in the basal short axis views tracking of the posterior mitral leaflet during diastole should be excluded to avoid artefacts. This problem might be solved by the analysis of at least two or multiple recordings of the same sectional plane including scanning from different transducer positions after changing the proband’s position or the intensity of the breathing maneuvre. Of course, if no reason for the pathologic waveform can be detected, and if these artefacts cannot be eliminated in full myocardial tracking of the LV wall, the resulting waveforms are less likely due to artefact tracking. Examples of inadequate LV tracking in the basal LV segments are structures like the atrial myocardium, the coronary sinus, and/or mitral valve annulus. All CS artefacts, which were observed in the basal LV segments in the present study, could be explained by oblique sectional planes and tracking of atrial myocardium or aortic root.

The analysis of LV rotation by 2DSTE depends on the standardization and correct LV levels of the acquired sectional short axis planes. Only short axis views, which are perpendicular to the central line of the LV long axis view, enable plausible results. However, the basal short axis view is easily to be checked for adequacy of standardization. The main problem acquiring the basal short axis views is the intersection of the mitral annulus and the left atrium. This problem can be solved by biplane scanning technique to document the correct perpendicular views. Thus, oblique scanning of the LV walls and too cranial or caudal transducer positions should be avoided. In addition, parasternal biplane scanning enables the standardization of apical short axis views to document the adequate LV levels for the assessment of apical LV rotation.

### The rationale to focus on CS, RS and LV rotation and layer-specific LV deformation patterns by 2DSTE

The involvement of different LV layers has been observed in various cardiac diseases, which were documented by pathognomonic patterns of late enhancment (LE) in cardiac magnetic resonance tomography (CMR) [24]. Thus, territoreal subendocardial LE is e.g. characteristic for ischemic heart disease, ubiquitous subendocardial LE e.g. for myocardial infarction with non-obstructive coronary arteries, territoreal transmural LE e.g. for sarcoidosis, homogeneous diffuse LE e.g. for storage diseases, and predominantly subepicardial LE e.g. for viral myocarditis [24, 39]. Despite the small amount of LV thickening by the outer LV layers the analysis of CS by 2DSTE was described as an useful tool to detect LV pathiologies [40–42]—especially LV involvement due to acute myocarditis [19–23]. The early diagnosis of acute myocarditis with normal or preserved LV ejection fraction might be one important target of CS and LV rotation analysis by 2DSTE. However, the patterns of myocarditis are obviously very heterogenoeous with respect to territorial as well as layer-specific involvement making the diagnosis more difficult using TTE [19, 20, 22, 23]. The echocardiographic detection of myocarditis might be of particular interest in special patient cohorts like athletes or professionals for passenger transport [21]. The risk of potential malignant arrhythmias due to acute myocardits during competetive sport or in the presence of high responsibilities might justify the approaches to improve diagnostic tools like echocardiography which is widely available. A second diagnostic target of CS and LV rotation analysis by 2DSTE might be the early detection of cardiotoxic alterations in cancer patients during chemotherapy and of regional abnormalities of LV deformation due to granulomatous or storage diseases [43–47].

The main diagnostic problem—especially in patients with acute myocarditis—is the regional correlation of CS artefacts with definite pathological findings of LE, which is most frequently seen in the basal posterior and lateral segments in CMR studies. Thus, the differentiation of 2DSTE artefacts from pathological findings is important and can only be solved by an accurate methodologic approach.

### Limitations

The present study was performed in healthy volunteers and in athletes who often have a good parasternal acoustic window. Thus, these cohorts do generally not represent the image quality of patients with cardiac diseases, in whom lung diseases, obesity and multiple other reasons cause limitations of image quality. However, with respect to the potential detection of myocarditis these cohorts are of particular interest for echocardiographic screening. It should be accentuated that the current study does not validate this algorithm to differentiate between athlete's heart and cardiomyopathies. This algorithm only provides a methodological guide to verify artefact detection by checking the strain waveforms with respect to plausibility. If 2DSTE of parasternal short axis views fails due to bad image quality, perhaps 3D strain analysis of 3D data sets with high image quality is fundamentally able to overcome the limitations of 2D TTE, but in comparison to 2D TTE, spatial and temporal resolution of 3D TTE is lower. So far, 3D STE is rarely tested—especially for the assessment of CS, RS and LV rotation [48, 49].

## Conclusions

The proposed algorithm seems to facilitate the differentiation between pathological strain and artifacts. 2DSTE of parasternal views might be a useful method to quantify RS, CS, and LV rotation. The prerequisites to apply this technique are the acquisition of representative standardized views and the full LV myocardial tracking excluding valvular or paraventricular structures. Biplane scanning is useful to verifiably document short axis views perpendicular to the LV long axis view, and to exclude oblique short axis views as well as artefacts due to paraventricular structures.The proposed algorithm for analysis of LV rotational deformation by 2DSTE helps to avoid artefacts with respect to the interpretation of deviant strain waveforms and might contribute to a standardization of this technique and will encourage the use of 2DSTE in the clinical scenario- The present study showed that the algorithm is feasible in healthy volunteers and athletes with adequate parasternal image quality. Thus, 2DSTE might provide an interesting diagnostic tool for the detection of viral myocarditis in athletes and in patients with an adequate parasternal image quality—especially at stages with normal or preserved LV ejection fraction.

## Supplementary Information

Below is the link to the electronic supplementary material.

Supplementary file1 (TIF 2251 kb)

Supplementary file2 (TIF 2147 kb)

Supplementary file3 (TIF 457 kb)

Supplementary file4 (TIF 836 kb)

Supplementary file5 (TIF 2407 kb)

Supplementary file6 (TIF 1228 kb)

Supplementary file7 (DOCX 53 kb)

Supplementary file8 (DOCX 14 kb)
