# Supplementary figures and images for: Analysis of left ventricular rotational deformation by 2D speckle tracking echocardiography: a feasibility study in athletes
Source: Int J Cardiovasc Imaging. 2021 Mar 18;37(8):2369–86. doi: 10.1007/s10554-021-02213-3 (PMC8302535; doi:10.1007/s10554-021-02213-3)

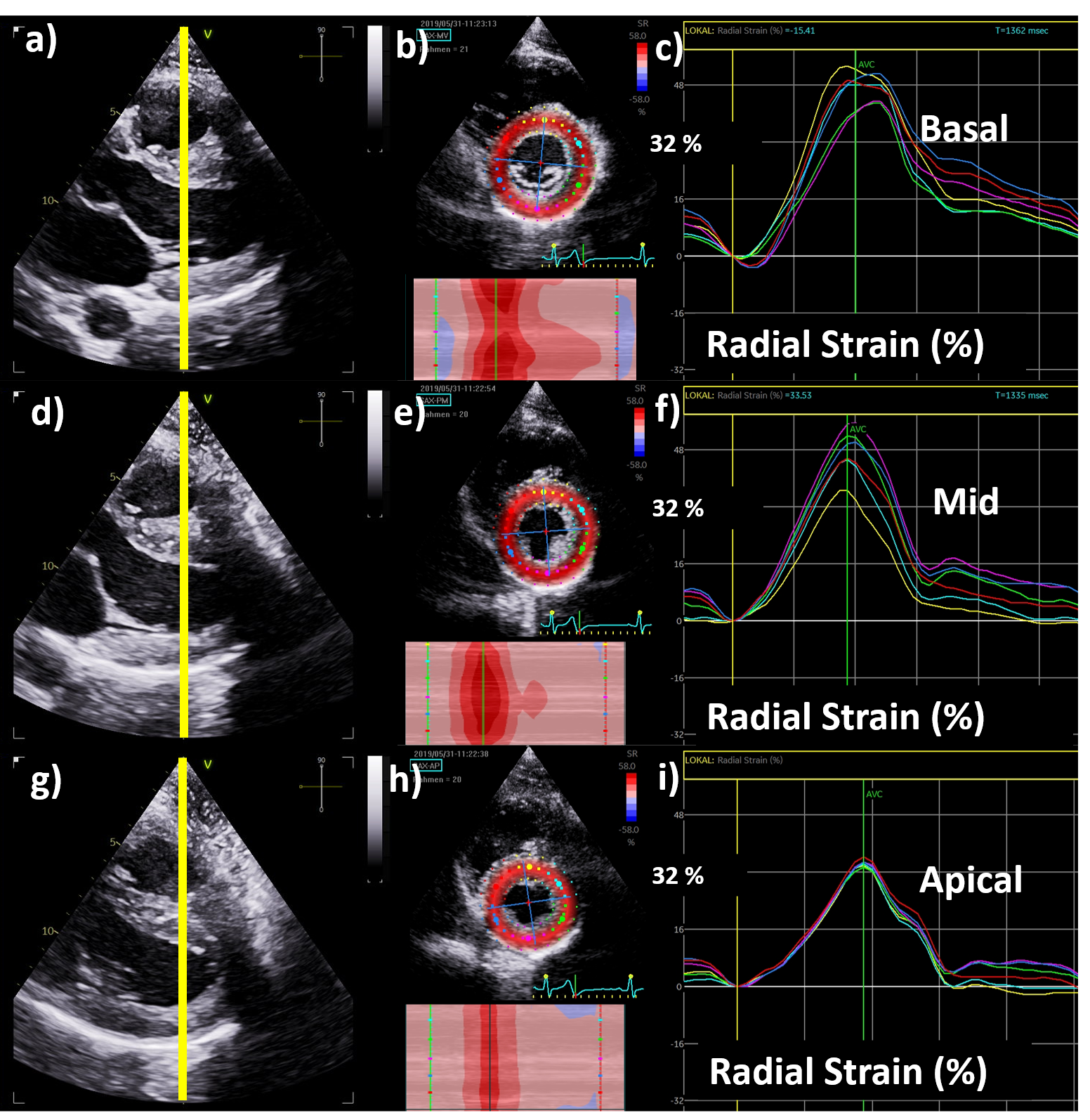

Supplement: Supplementary file 1 — Supplementary file1 (TIF 2251 kb) [file 10554_2021_2213_MOESM1_ESM.tif]

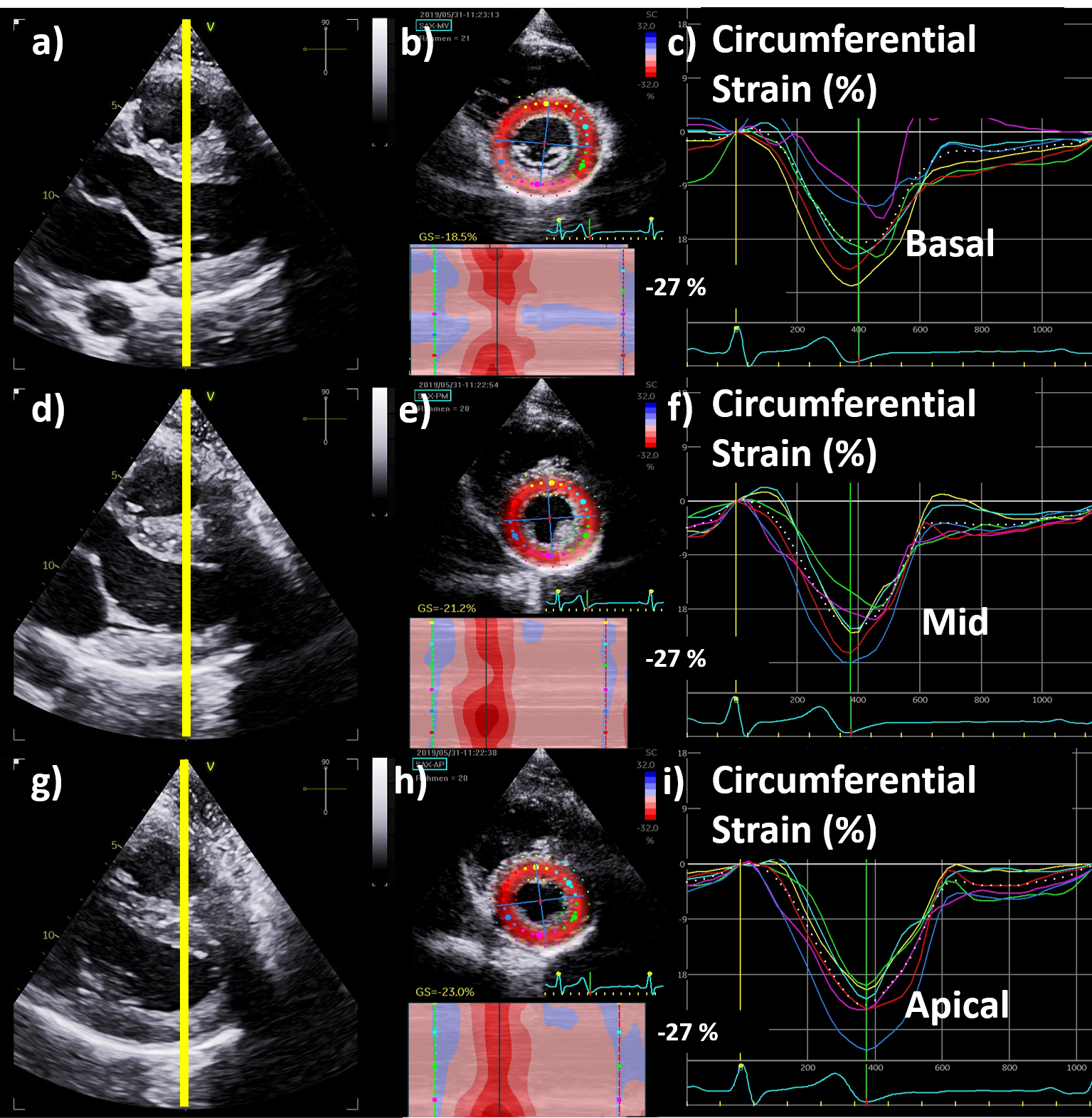

Supplement: Supplementary file 2 — Supplementary file2 (TIF 2147 kb) [file 10554_2021_2213_MOESM2_ESM.tif]

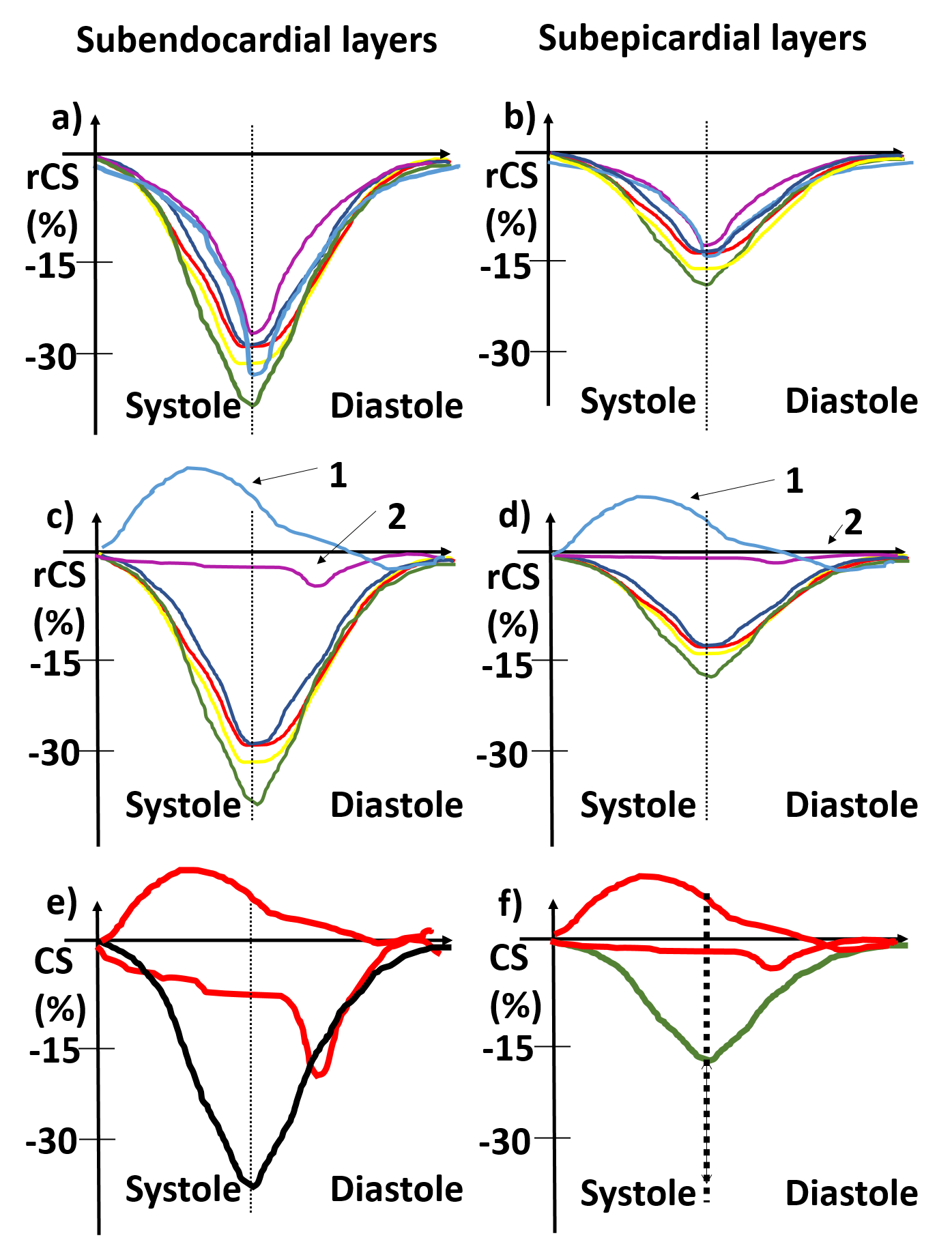

Supplement: Supplementary file 3 — Supplementary file3 (TIF 457 kb) [file 10554_2021_2213_MOESM3_ESM.tif]

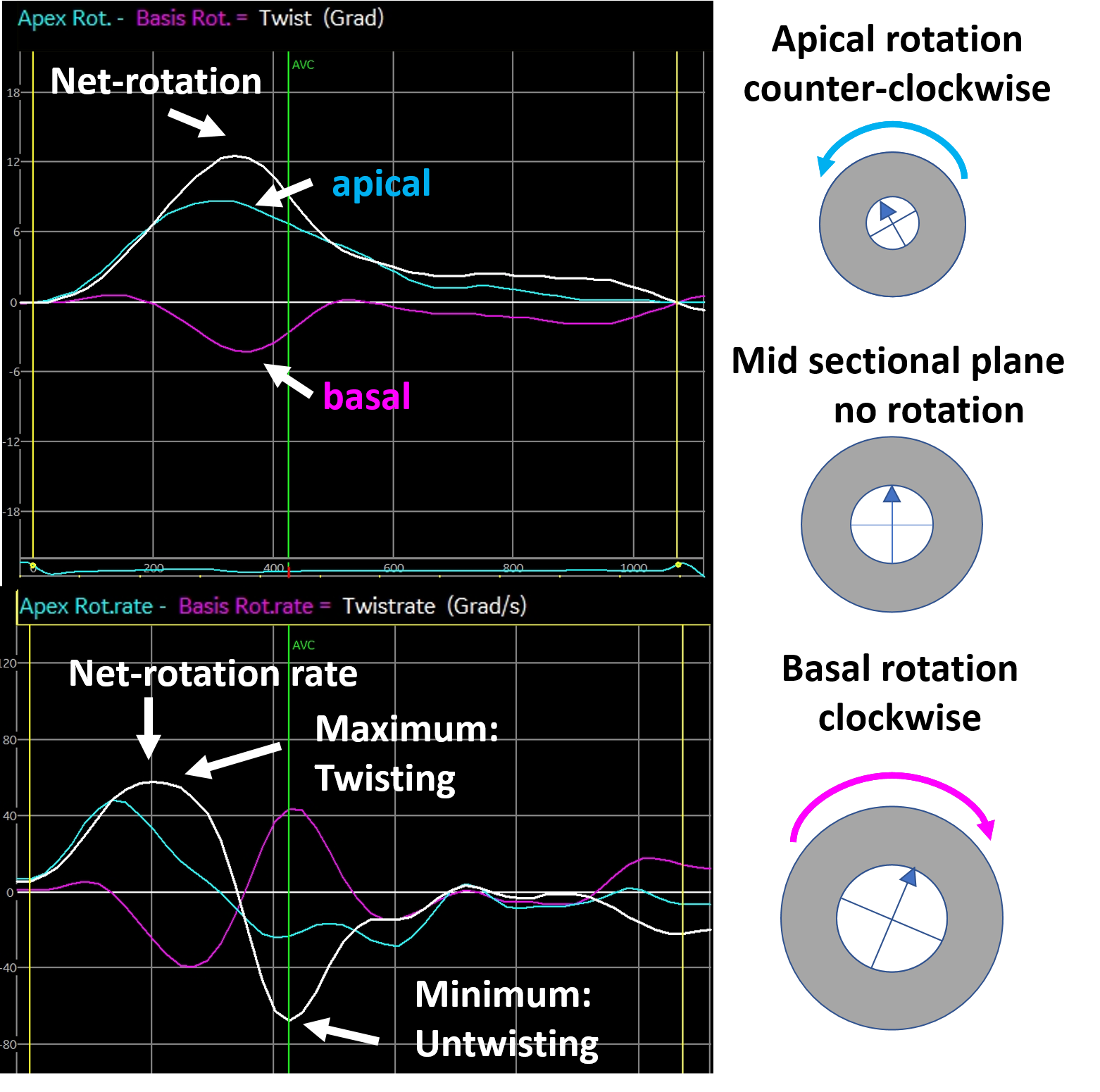

Supplement: Supplementary file 4 — Supplementary file4 (TIF 836 kb) [file 10554_2021_2213_MOESM4_ESM.tif]

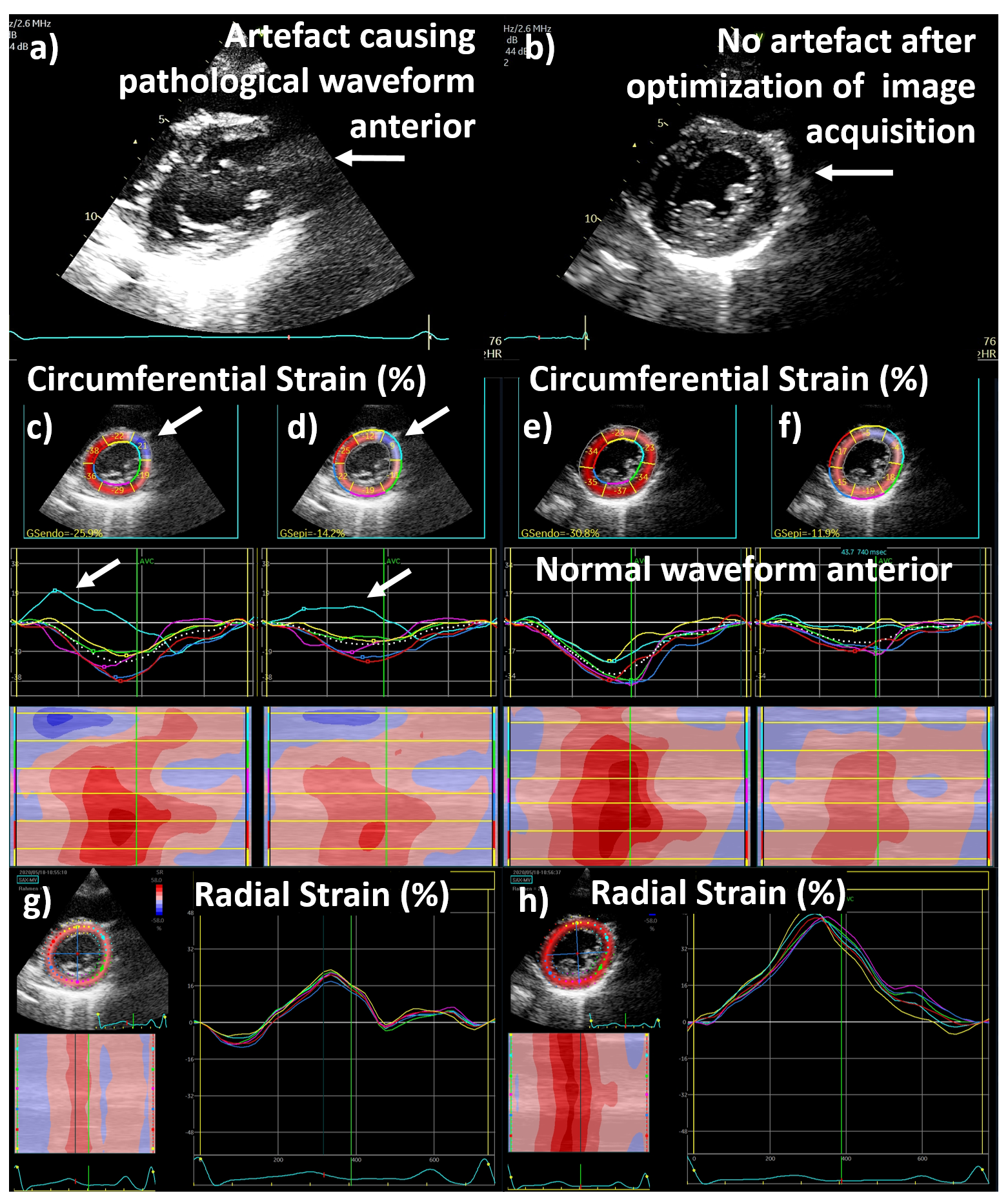

Supplement: Supplementary file 5 — Supplementary file5 (TIF 2407 kb) [file 10554_2021_2213_MOESM5_ESM.tif]

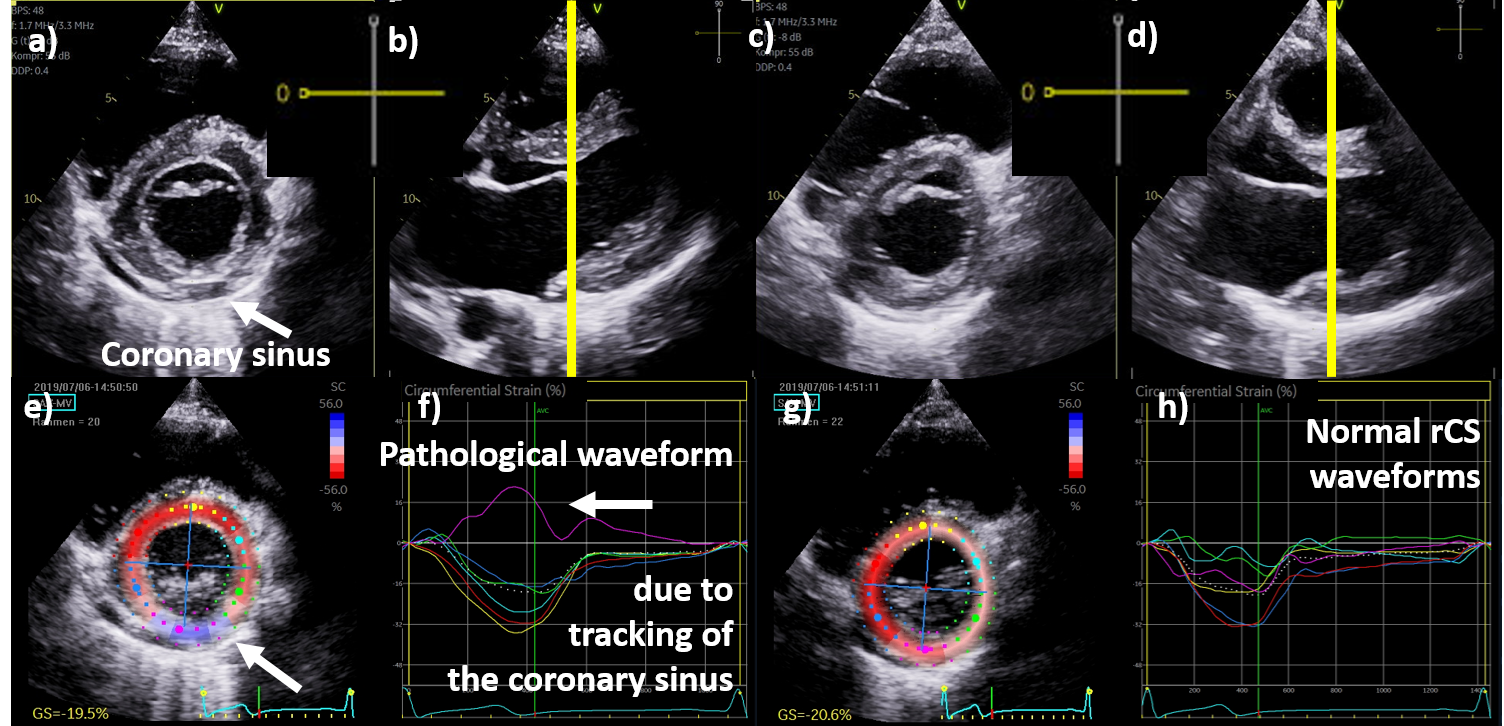

Supplement: Supplementary file 6 — Supplementary file6 (TIF 1228 kb) [file 10554_2021_2213_MOESM6_ESM.tif]
