# Supplementary material for: Analysis of left ventricular rotational deformation by 2D speckle tracking echocardiography: a feasibility study in athletes
Source: Int J Cardiovasc Imaging. 2021 Mar 18;37(8):2369–86. doi: 10.1007/s10554-021-02213-3 (PMC8302535; doi:10.1007/s10554-021-02213-3)
Supplement: Supplementary file 7 — Supplementary file7 (DOCX 53 kb) [file 10554_2021_2213_MOESM7_ESM.docx]

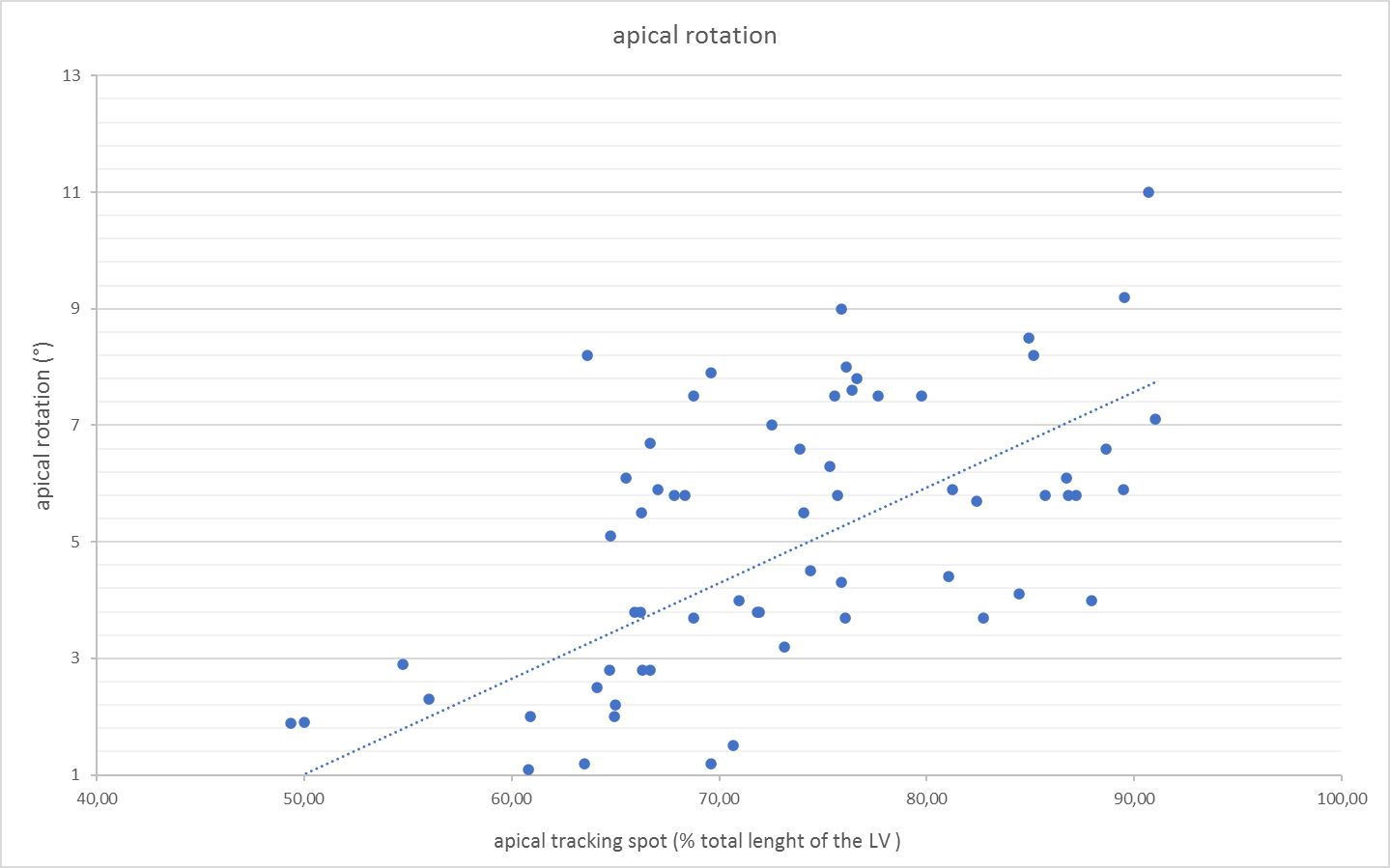


40 %

50 %

60 %

70 %

80 %

90 %

100 %

**Relation between apical LV rotation and LV level of corresponding short axis views**

**Apical LV rotation (°)**

1°

3°

5°

7°

9°

11°

**Level of apical short axis view (% of total LV long axis)**

**Y= 0.1589x • 6.77265**

**R^2^ = 0.3944**
