# Supplementary material for: Analysis of left ventricular rotational deformation by 2D speckle tracking echocardiography: a feasibility study in athletes
Source: Int J Cardiovasc Imaging. 2021 Mar 18;37(8):2369–86. doi: 10.1007/s10554-021-02213-3 (PMC8302535; doi:10.1007/s10554-021-02213-3)
Supplement: Supplementary file 8 — Supplementary file8 (DOCX 14 kb) [file 10554_2021_2213_MOESM8_ESM.docx]

Figures legends

**Figure 1:** Illustration of physiological waveform patterns of radial strain (RS). Long axis view during systole (a) with the corresponding basal short axis view and the respective color M-Mode of regional RS (b) and the corresponding RS curves (c) - the yellow line in (a) shows the level of the sectional plane in (b); long axis view during systole (d) with the corresponding mid short axis view and the respective color M-Mode of regional RS (e) and the corresponding RS curves (f) - the yellow line in (d) shows the level of the sectional plane in (e); long axis view during systole (g) with the corresponding apical short axis view and the respective color M-Mode of regional RS (h) and the corresponding RS curves (i) - the yellow line in (g) shows the level of the sectional plane in (h).

**Figure 2:** Illustration of physiological waveform patterns of circumferential strain (CS). Long axis view during systole (a) with the corresponding basal short axis view and the respective color M-Mode of regional CS (b) and the corresponding CS curves (c) - the yellow line in (a) shows the level of the sectional plane in (b); long axis view during systole (d) with the corresponding mid short axis view and the respective color M-Mode of regional CS (e) and the corresponding CS curves (f) - the yellow line in (d) shows the level of the sectional plane in (e); long axis view during systole (g) with the corresponding apical short axis view and the respective color M-Mode of regional CS (h) and the corresponding CS curves (i) - the yellow line in (g) shows the level of the sectional plane in (h).

**Figure 3:** Scheme of regional and global circumferential strain (rCS; CS) curves: rCS and CS curves of subendocardial layers are displayed at the left side, the curves of subepicardial layers on the right side. Normal waveforms are displayed in (a) and (b). Pathological regional waveforms with dyskinetic (1) and hypokinetic (2) waveforms are displayed in (c) and (d). The normal global CS curve of the subendocardial layers (e - black) shows a larger minimum amplitude than the CS curve of the subepicardial layers (f - green); the red lines in (e) and (f) represent pathological CS waveforms.

**Figure 4:** Illustration of apical and basal left ventricular rotation. The basal clockwise rotation (magenda) and the apical counter-clockwise rotation (blue) is presented on the left side as graphs of rotation and rotation rate and on the right side by arrows (scheme). The white waveforms represent net-rotation and net-rotation rate causing left ventricular twisting and untwisting.

**Figure 5:** Illustration of artefact tracking caused by missing contact between the transducer and the patient’s skin. Obviously, a visible artefact (a - white arrow) causes a deviation of the circumferential strain (CS) waveform of the anterior LV segment (a) at the level of the papillary muscle (mid parasternal short axis view). This artefact could be eradicated by scanning optimization (b). CS analysis showed dyskinesia in the color-coded M-Mode as well as in the regional waveforms (c - subendocardial layer (white arrows); d - subepicardial layer (white arrows)). No artefacts are observed if CS analysis was performed by optimized cineloops (e, f). The shape of radial strain (RS) curves was not altered in this example. However, the analysis of images with insufficient quality (g) resulted in significant lower peak maximum RS values in comparison to optimized cineloops (h).

**Figure 6:** Illustration of artefact tracking by non-myocardial structures. Biplane illustration of a basal short axis view (a) with the corresponding long axis view (b). The yellow line in (b) displays the sectional plane of the corresponding short axis view in (a). Biplane scanning documents an oblique sectional plane with the intersection of the coronary sinus within the cardiac cycle (a,b) showing dyskinesia (e – white arrow) and a pathological waveform (f) in the posterior region. After optimization of image acquisition (c,d) the intersection of the coronary sinus was avoided. Full myocardial tracking of the left ventricular wall within the cardiac cycle shows nearly physiological color-coded patterns (g) and waveforms (h).

**Figure 7:** Relation between apical left ventricular (LV) rotation and the level of the corresponding short axis view: The x-coordinate represents the level of the apical short axis view as percentage of the total length of the left ventricle during end systole. The y-coordinate represents the degree of apical LV rotation determined in the respective sectional plane. Obviously, a presumably physiological apical counter-clockwise LV rotation of > 3° can only be observed within the apical third of the left ventricle.
